# Experimental workflows for the accurate identification of mitochondrial redox events

**DOI:** 10.1016/j.redox.2026.104387

**Published:** 2026-09-09

**Authors:** Pablo Hernansanz-Agustín, Juan Pedro Bolaños, Consuelo Borrás, Elisa Cabiscol, Victoria Campuzano, Laura de Cubas, José Antonio Enríquez, Isabel Fabregat, Gemma Gonfaus-Ortiz, Elena Hidalgo, Marc Liesa, Cristina Mas-Bargues, Antonio Martínez-Ruiz, María Monsalve, Rubén Quintana-Cabrera, Sergio Rius-Pérez, Juan Sastre, Antonio Zorzano

**Affiliations:** a Centro de Neurociencias Cajal (CNC), Consejo Superior de Investigaciones Científicas (CSIC), Avenida de León 1, Alcalá de Henares, 28805, Spain; b Institute of Functional Biology and Genomics, University of Salamanca, CSIC, Salamanca, 37007, Spain; c Centro de Investigación Biomédica en Red Fragilidad y Envejecimiento Saludable-Instituto de Salud Carlos III (CIBERFES-ISCIII), Spain; d Institute of Biomedical Research of Salamanca, University Hospital of Salamanca, University of Salamanca, CSIC, Salamanca, 37007, Spain; e MiniAging/Freshage Research Group, Department of Physiology, Faculty of Medicine, University of Valencia, Institute of Health Research-INCLIVA, Valencia, 46010, Spain; f Dept. Ciències Mèdiques Bàsiques, Facultat de Medicina, Universitat de Lleida, Av. Alcalde Rovira Roure 80, Lleida, 25198, Spain; g Institut de Recerca Biomèdica de Lleida Fundació Dr. Pifarré (IRBLleida), Av. Alcalde Rovira Roure 80, Lleida, 25198, Spain; h Department de Biomedical Sciences, School of Medicine and Health Sciences, University of Barcelona, Barcelona, 08036, Spain; i Institut d'Investigacions Biomèdiques August Pi i Sunyer (IDIBAPS), Barcelona, 08036, Spain; j Centro de Investigación Biomédica en Red Enfermedades Raras-Instituto de Salud Carlos III (CIBERER-ISCIII), Spain; k Oxidative Stress and Cell Cycle Group, Universitat Pompeu Fabra, C/ Dr. Aiguader 88, 08003, Barcelona, 08908, Spain; l Cardiovascular Regeneration Program, Centro Nacional de Investigaciones Cardiovasculares (CNIC), Madrid, 28029, Spain; m TGF-β and Cancer Group, Bellvitge Biomedical Research Institute (IDIBELL), L'Hospitalet de Llobregat, Barcelona, 08908, Spain; n Centro de Investigación Biomédica en Red Enfermedades Hepáticas y Digestivas-Instituto de Salud Carlos III (CIBERHED-ISCIII), Spain; o Institut de Biologia Molecular de Barcelona (IBMB) CSIC, Barcelona, Spain; p CIBERDEM, Instituto de Salud Carlos III, Madrid, Spain; q Unidad de Investigación, Hospital Universitario Santa Cristina, Instituto de Investigación Sanitaria Princesa (IIS-Princesa), Madrid, 280009, Spain; r Instituto de Investigaciones Biomédicas Sols-Morreale (CSIC-UAM), Arturo Duperier, 4, Madrid, 28029, Spain; s Department of Cell Biology and Functional Biology, Faculty of Biology, University of Valencia, Valencia, Spain; t Department of Physiology, Faculty of Pharmacy & Food Sciences, University of Valencia, Avda. Vicente Andrés Estellés 22, Burjasot, Valencia, 46100, Spain; u Institute for Research in Biomedicine (IRB Barcelona), Department of Biochemistry and Molecular Medicine, Universitat de Barcelona, Barcelona, 08028, Spain

**Keywords:** Workflow, Redox event, Mitochondrial ROS, ROS probe, Antioxidant, (Patho)physiology

## Abstract

The study of redox biology has been growing constantly since the last decades. Over these years, redox processes have been linked to an extraordinarily wide range of physiological and pathological events, becoming recognized as central mechanisms underlying many of them. In this context, it becomes essential to understand the advantages and limitations of the tools under use, to recognize the specific controls required for each measurement and to accurately distinguish between distinct redox mechanisms. So far, multiple and excellent reviews have dealt with either the tools, the protocols or the mechanisms involved in reactive oxygen species (ROS) production and quenching, a.k.a. redox events. However, a review outlining the *workflows* to appropriately detect them is still lacking. We define workflow as the combination of tools, methods and mechanistic knowledge that allow the definition of a specific redox event. In this review, we aim to provide an optimal workflow for the research on mitochondrial redox events. To this end, we first summarize the molecular tools available to measure and quench ROS. We then explain the mechanisms of ROS production and scavenging in several of the cellular compartments, with special focus on mitochondria, as well as their implication in physiology and disease. Finally, we use the knowledge in all sections to build a recommended experimental workflow, illustrated by several cases of study. This review will enable the reader to understand how specific mitochondrial redox events can be accurately measured, considering all technical, methodological and mechanistical variables and limitations required for their reliable detection and interpretation.

## 1 Introduction

All metazoans require oxygen (O_2_) for survival, as it is essential for both energy transduction and cellular signaling. The stepwise reduction of molecular O_2_ generates reactive O_2_ species (ROS). A one-electron reduction of O_2_ produces the superoxide anion (O_2_^•-^), the most common initial intermediate generated by ROS-producing enzymes and a highly reactive species. O_2_^•-^ is short-lived and largely confined to its site of generation. Subsequent one-electron reduction or dismutation (either spontaneous or enzyme-catalyzed) of O_2_^•-^ yields hydrogen peroxide (H_2_O_2_), a well-characterized ROS that often functions as a second messenger owing to its ability to reversibly oxidize protein thiol groups, its relative stability, and its capacity to diffuse over short distances or be transferred through relay mechanisms [[1], [2], [3]]. Further one-electron reduction of H_2_O_2_ generates the hydroxyl radical (^•^OH), primarily through the so-called Fenton reaction, producing an extremely damaging ROS. Although this sequence provides a general framework, several enzymes can generate H_2_O_2_ directly. In parallel, ROS can be quenched by antioxidant systems, which are capable of eliminating all the aforementioned ROS species. Throughout this review, we use the term redox events to refer collectively to both the production and quenching of ROS.

Given the short-lived nature and high reactivity of ROS, their measurement is particularly challenging. Moreover, redox events vary according to the cellular microenvironment to such an extent that the same molecule may either inhibit or promote ROS production depending on the metabolic conditions of a specific subcellular compartment. Consequently, it is essential for researchers to understand both the advantages and limitations of the molecular tools employed and how experimental conditions may influence the results obtained. As a result, the use of a single tool or protocol is generally insufficient to accurately measure a specific redox event. Instead, the combined application of multiple complementary or orthogonal approaches is typically required to reliably identify and characterize a given redox process. This integrated strategy is what we define as a workflow. The aim of this review is to provide readers with the knowledge necessary to design and implement their own experimental workflows, an increasingly important objective in a field experiencing growing interest. Indeed, a simple PubMed search using the terms “redox biology” reveals a marked increase in their prevalence within the scientific literature, rising from 191 publications in 2000 to 2761 publications in 2025. We believe that equipping readers with the knowledge required to technically and mechanistically dissect specific mitochondrial redox events will substantially improve data interpretation, deepen our understanding of the role of ROS in physiology and pathology, and ultimately enhance the impact of future studies dedicated to this matter.

This review progresses from the basic tools and fundamental principles of redox biology to practical strategies for dissecting redox events in complex biological systems. To this end, we first describe the molecular tools available for measuring and manipulating mitochondrial ROS (mtROS), including both genetic and chemical approaches, thereby enabling the selection of the most appropriate strategy for the accurate assessment of specific redox events. We then provide an integrated overview of several ROS-generating systems located in the cytosol and non-mitochondrial organelles, including NADPH oxidases (NOXs), xanthine oxidoreductase (XOR), peroxisomes, endoplasmic reticulum (ER) oxidoreductases, and monoamine oxidases (MAOs). Providing an overview of non-mtROS sources will highlight the multifactorial nature of cellular redox biology and will allow the explanation of several concepts in section 4.2. Given the multifactorial nature of mtROS production, we also examine how mtROS generation is influenced by bioenergetic state and cellular metabolism, as well as its roles in signaling and disease. In addition, we describe the mitochondrial antioxidant systems and the metabolic and transcriptional mechanisms that regulate them. Finally, building upon the concepts introduced throughout the review, we propose several experimental workflows designed to identify specific redox events through the integration of technical, methodological, and mechanistic variables, thereby enabling their rigorous assessment and interpretation.

## 2 Tools to measure and manipulate ROS levels

Because ROS are highly reactive and short-lived, their accurate detection remains a major challenge. In this section, Because ROS are highly reactive and short-lived, their accurate detection remains a major challenge. In this section, we discuss the tools currently available to address this issue. Two of the principal challenges in redox biology are the identification of methods capable of detecting H_2_O_2_ or O_2_^•-^ with high efficiency and specificity, and the determination of the subcellular location in which ROS fluctuations occur. Addressing these challenges requires real-time monitoring in living cells. With regard to H_2_O_2_ detection, Section 2.1 discusses how these requirements are best fulfilled by genetically encoded biosensors, particularly peroxiredoxin (PRX)-coupled roGFP probes and HyPer variants, which provide the highest spatial and temporal resolution currently available for H_2_O_2_ measurements.

Several existing methods for ROS quantification rely on redox-active fluorescent or luminescent probes, such as dihydrodichlorofluorescein (DCFH_2_), which continue to form the basis of many commercial detection kits. However, these probes present important limitations and can frequently lead to misinterpretation of results [4]. Indeed, it has recently been demonstrated that fluorescein-based dyes in bacteria do not function as ROS reporters; rather, they reflect cellular energy depletion, which slows the activity of dye efflux pumps and results in intracellular dye accumulation [5]. In Section 2.2, we further examine these limitations and provide recommendations and alternative approaches for the use of fluorescent probes.

Finally, in Section 2.3, we evaluate complementary strategies for ROS quenching that can be used to validate or challenge changes in ROS levels inferred from protein-based biosensors or small-molecule fluorescent probes.

This section, as depicted in Section 6, is the building basis to design experimental workflows since it enables the reader to select the tools to use in their experimental design. Together with the knowledge in sections 3, 4.1, 5, this section provides with the necessary technical and mechanistical understanding to generate workflows (as sections 4.2, 4.3 reinforce the methodological and conceptual grounds experimental workflows).

### 2.1 Genetically encoded reporters of H_2_O_2_

To overcome the limitations of cell-permeable fluorescent dyes, the scientific community has developed a range of protein-based reporters. These reporters predominantly belong to two families: HyPer derivatives [6] and the roGFP-based sensors [7], in which roGFP is engineered as a fusion with peroxidase enzymes [[8], [9], [10]]. Both HyPer and roGFP biosensors enable ratiometric detection of H_2_O_2_ through dual-excitation fluorescence changes driven by redox-dependent conformational shifts. Specifically, H_2_O_2_-dependent oxidation of these biosensors via disulfide bond formation induces conformational changes that alter their fluorescence properties at two excitation wavelengths (405 nm and 488 nm). The fluorescence emitted at 520 nm following excitation at these wavelengths can then be used to calculate the degree of probe oxidation and infer relative H_2_O_2_ levels [11].

**Fig. 1:**
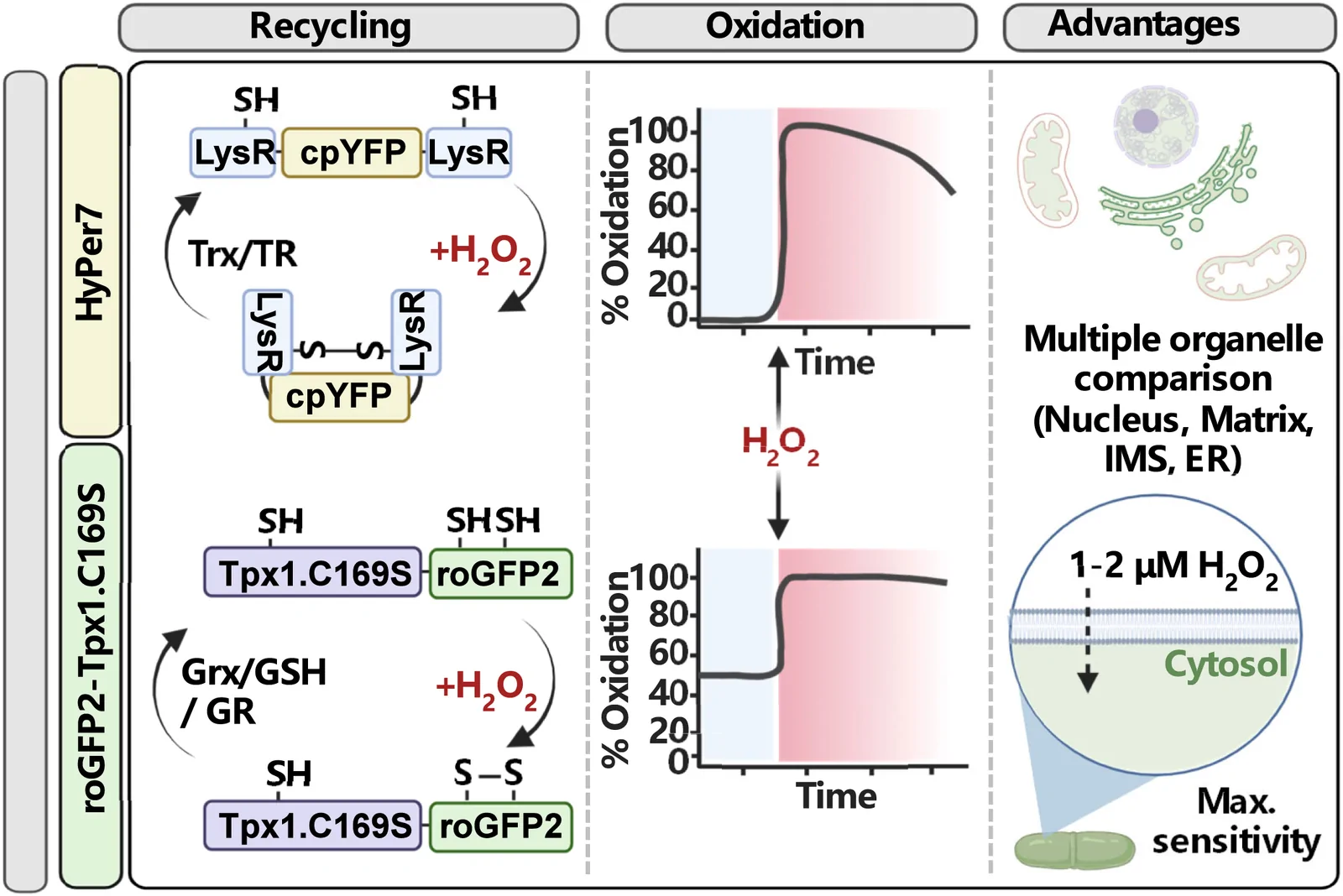
**Schematic comparison of genetically encoded H_2_O_2_ biosensors.** Redox biosensors to measure H_2_O_2_ include members of the HyPer family (HyPer7) and roGFP2 derivatives fused to PRXs (roGFP2–Tpx1.C169S). From left to right, the main differences between these biosensors are their recycling by distinct electron donor systems, their oxidation percentage under steady-state conditions, and their specific advantages. HyPer7 is constructed by fusion of a LysR transcriptional domain to a circularly permuted YFP (cpYFP). It is recycled by the TRX/TRX reductase (TRXR) system, which, together with its lower sensitivity, results in a mostly reduced biosensor under steady-state conditions. These properties allow HyPer7 to detect H_2_O_2_ fluctuations in different compartments, including the nucleus, mitochondrial matrix, mitochondrial intermembrane space (IMS), and ER. roGFP2–Tpx1.C169S is generated by fusion of PRX Tpx1.C169S (point mutation of the resolving cysteine) to the redox-sensitive GFP, roGFP2. It is recycled by the GRX/GSH/GR system. In combination with its high sensitivity, roGFP2–Tpx1.C169S shows ∼50% basal oxidation and can detect extracellular H_2_O_2_ concentrations of ∼1–2 μM.

In this section, we highlight the advantages of three such sensors (roGFP2-Tpx1.C169S, HyPer, and HyPer7) and explain how straightforward mathematical and comparative analyses of biosensor oxidation kinetics can be used to quantify H_2_O_2_ gradients across membranes and steady-state peroxide levels in eukaryotic systems. Fluorescence measurements can be performed using a variety of experimental platforms, including flow cytometry, confocal fluorescence microscopy, and fluorescence plate readers. Importantly, measurements should be conducted under physiologically relevant conditions, as cellular metabolism and ROS production and scavenging can vary substantially in response to factors such as cell density, metabolic activity, O_2_ tension, temperature, and pH. In the following sections, we compare these three sensors to facilitate the selection of the most appropriate probe for specific experimental applications, with special focus on the sensitive HyPer7 and roGFP2-Tpx1.C169S (Fig. 1):

a) Mechanism of action, sensitivity, and suitability: HyPer is derived from the Gram-negative bacterial protein OxyR, with a circularly permuted YFP (cpYFP) inserted between the redox-active cysteines of the OxyR transcription factor [6]. The original HyPer represented one of the first genetically encoded sensors for H_2_O_2_ detection; however, its performance was strongly influenced by pH fluctuations. The recently developed HyPer7, derived from *Neisseria meningitidis* OxyR, is largely pH-insensitive and exhibits substantially greater sensitivity than the original sensor [11,12]. In contrast, roGFP relies on engineered cysteine residues that form disulfide bonds upon oxidation. Its sensitivity has been markedly enhanced through fusion to peroxidase-based modules, such as Orp1 [13] or PRXs (e.g., Tpx1.C169S) [8], enabling the detection of very low intracellular H_2_O_2_ concentrations.

Both roGFP2-Tpx1.C169S and HyPer7 can monitor nanomolar fluctuations in intracellular H_2_O_2_, with roGFP2-Tpx1.C169S being approximately two-to fivefold more sensitive than HyPer7. Under basal conditions, cytosolic roGFP2-Tpx1.C169S is partially oxidized, whereas HyPer7 remains almost fully reduced. Comparative studies in fission yeast have revealed detection thresholds of approximately 100 μM extracellular H_2_O_2_ for HyPer, 5–10 μM for HyPer7, and 1–2 μM for roGFP2-Tpx1.C169S. Consequently, the original HyPer is poorly suited for monitoring early signaling events, whereas HyPer7 and roGFP2-Tpx1.C169S operate within the physiological range of peroxide fluctuations. Overall, sensor selection depends on the sensitivity required and the cellular compartment under investigation. HyPer7 is particularly suitable for mitochondrial measurements, whereas roGFP2-Tpx1.C169S appears to lose responsiveness within this organelle [11] (personal communication).

b) Reduction system: The reduction mechanisms also differ between the two biosensor families. Whereas the recovery kinetics of HyPer7 reflect thioredoxin (TRX)-dependent reduction capacity in both mammalian and yeast cells [11,14], roGFP recycling depends on the glutathione–glutaredoxin (GSH–GRX) system [8]. Consequently, when the basal oxidation state of a biosensor is used as a proxy for intracellular H_2_O_2_ levels, it is important to recognize that the signal reflects not only peroxide abundance but also the activity of the corresponding reduction pathway.

These protein biosensors have been successfully expressed in a wide range of eukaryotic model systems, including cultured cells [12,15], organs or whole organisms [16,17]. In many cases, the spatiotemporal detection of H_2_O_2_ fluxes has been combined with the chemogenetic generation of peroxides through d-amino acid oxidase (DAO). Differential compartment-specific expression of HyPer7 and DAO enables the investigation of signaling and toxic effects associated with transmembrane peroxide diffusion [18], providing a powerful approach for studying interorganellar signaling and redox communication.

In conclusion, we strongly recommend the use of genetically encoded reporters for monitoring peroxide dynamics in living cells. Both HyPer7 and roGFP2-Tpx1.C169S provide sufficient sensitivity to detect moderate, signaling-relevant fluctuations in cytosolic H_2_O_2_. However, only HyPer7 retains robust functionality within mitochondria, making it the preferred sensor for investigating mitochondrial peroxide dynamics.

On the other hand, it should be noted that a major limitation of genetically encoded H_2_O_2_ biosensors is that their use in whole mammalian organisms generally requires surgical tissue exposure. Nevertheless, recent advances have enabled the expression of roGFP-based probes in organs such as the kidney, where redox changes can be monitored by two-photon microscopy following exposure of the intact organ [19].

### 2.2 Small fluorescent dyes to measure ROS

Small fluorescent dyes are widely used to measure ROS in cell-free systems, cultured cells, and whole organisms, and are compatible with a variety of detection platforms, including flow cytometry and fluorescence microscopy. Their fluorescence generally increases upon oxidation, often in an irreversible manner. Because the steady-state level of any ROS species reflects the balance between its production and removal, the signal generated by these probes reports net ROS abundance rather than ROS production itself. Several limitations should be carefully considered before selecting a fluorescent probe:

a) Specificity. Many probes react with multiple oxidants, including reactive species that are not classically classified as ROS, complicating the attribution of fluorescence signals to a single oxidant species. Consequently, the chemical reactivity and specificity of each probe must be critically evaluated before experimental use.
b) Quantification and controls. Semi-quantitative measurements require appropriate positive and negative controls within the relevant concentration range. These controls should be established by experimentally modulating ROS production or clearance. Both technical controls, which assess methodological variability such as pipetting accuracy or instrument performance, and biological controls, which account for variability among biological samples, are essential for reliable data interpretation.
c) Probe localization and retention. Certain probes can be actively exported from cells. For example, fluorescein-based dyes are efficiently removed from the bacterial cytoplasm through efflux systems [5]. Therefore, verification of intracellular localization and retention represents a critical preliminary control for any probe-based experiment [20].

When these limitations are properly considered, fluorescent probes can provide robust and informative data. Nevertheless, probe specificity often requires validation using orthogonal methodologies based on distinct detection principles, as extensively discussed in several consensus guidelines and methodological reviews [[20], [21], [22]].

Below, we summarize several practical considerations for the measurement of different ROS species (Table 1):

a) Detection of O_2_^•-^. Dihydroethidium (DHE, also known as hydroethidine, HE) and its mitochondria-targeted derivative MitoSOX are among the most widely used probes for O_2_^•-^ detection. Reaction with O_2_^•-^ generates the specific fluorescent product 2-hydroxyethidium; however, nonspecific oxidation generates ethidium, which exhibits overlapping fluorescence properties. Both products intercalate into DNA, further enhancing fluorescence intensity. Consequently, unequivocal detection of O_2_^•-^ requires chromatographic separation and quantification of the 2-hydroxyethidium product [23]. In microscopy and flow cytometry applications, selectivity can be improved by using excitation wavelengths around 490 nm (below 500 nm) and emission detection between 560 and 570 nm^24^, as well as by verifying minimal DNA-dependent signal amplification.

**Table 1:** Small molecules measuring ROS, sytems in which they can be measured and their controls.

Type of ROS	Probe	Method	Cross-reaction	System	Technical controls for specificity
O_2_^•−^	Dihydroethidine	Fluorescence microscopy	H_2_O_2_, other oxidants and DNA	Intact cells and mitochondria	SOD
	Dihydroethidine (or MitoSOX)	HPLC	-	Intact cells and mitochondria	SOD
	MitoSOX	Fluorescence microscopy	H_2_O_2_, other oxidants and DNA	Intact cells and mitochondria	SOD and measurement of ΔΨmt (check mitochondrial staining).
	MitoNeoD	Fluorescence microscopy	H_2_O_2_ and other oxidants	Intact organs and intact cells	SOD and measurement of ΔΨmt
	TEMPOL, mitoTEMPO, nitryl CT-03, DIPPMPO and cyclic hydroxylamines CMH	Electron paramagnetic resonance (EPR)	Any other radical	Tissue or cell homogenates	SOD
H_2_O_2_	Amplex Red	Fluorimetry	Presence of carboxylesterases, ONOO^−^ and other peroxides	Homogenates, intact cells and mitochondria (impermeable)	Catalase
	Boronate probes	Fluorescence microscopy or mass spectrometry	ONOO^−^ and other peroxides	Whole organism, intact organ, cell and mitochondria	Catalase
General ROS detectors	Dichlorofluorescein and derivates (CellRox, etc)	Fluorescence microscopy	Any type of oxidants, pH and ionic fluxes	Intact cells and mitochondria	-

The mitochondrial accumulation of MitoSOX depends on its triphenylphosphonium (TPP^+^) moiety and therefore on ΔΨmt. Parallel assessment of ΔΨmt is consequently essential for correct interpretation [24]. Moreover, prolonged incubation may result in probe redistribution, including apparent nuclear localization. For this reason, short incubation times and appropriate ΔΨmt controls are strongly recommended [25,26].

Mitochondrial aconitase activity can also serve as an indirect but highly sensitive indicator of mitochondrial O_2_^•-^ production, as its catalytic iron–sulfur (Fe/S) cluster is rapidly and reversibly inactivated by oxidative damage.

b) General ROS detection. DCFH_2_, most commonly used in its membrane-permeable diacetate form (DCFH_2_-DA), is oxidized intracellularly to generate the fluorescent product 2′,7′-dichlorofluorescein (DCF). However, this process lacks chemical specificity. Neither H_2_O_2_ nor peroxynitrite (ONOO^−^) directly oxidizes DCFH_2_; instead, oxidation is mediated by secondary radical intermediates. Consequently, DCF fluorescence reflects a composite signal arising from both ROS-dependent and ROS-independent processes and is influenced by factors such as O_2_ tension, redox-active metals, and pH. Careful controls and validation through orthogonal approaches are therefore essential [20].

Global reversible cysteine oxidation can also be used as a surrogate measure of ROS-dependent two-electron oxidation events. In addition to the genetically encoded sensors described in section 2.1, these modifications can be assessed using “redox-switch” methodologies. These approaches involve blocking reduced thiols, selectively reducing oxidized thiols (for example, with DTT), and subsequently labeling the newly exposed thiols with fluorescent or biotinylated reagents for detection following electrophoretic separation.

c) Detection of peroxides. Boronate-based probes detect H_2_O_2_ and ONOO^−^ through oxidation to phenolic products, enabling both fluorescence- and mass spectrometry-based readouts. Representative examples include the MitoB/MitoP system, which has been widely used to quantify mitochondrial peroxide production *in vivo*. Although these probes are often considered selective for peroxides, ONOO^−^ reacts substantially faster than H_2_O_2_ and may dominate probe oxidation under conditions of inflammation or nitrosative stress. This limitation should be carefully considered when interpreting experimental results. In parallel, given that the MitoB/MitoP system is also ΔΨmt-dependent, a careful examination of this parameter should be always assayed, considering that the results may result from differences in metabolic states [27].

Horseradish peroxidase (HRP)-based assays, such as Amplex Red, are widely employed to quantify extracellular H_2_O_2_ released from intact cells, isolated mitochondria, or enriched enzyme preparations [28]. Because these probes are membrane-impermeable, they do not provide information regarding compartmentalized intracellular ROS production. Moreover, HRP activity can be inhibited by compounds such as N-acetylcysteine (NAC) or by O_2_•^-^ itself, an effect that can often be prevented by the addition of SOD.

d) Detection of radical species. Electron spin resonance/electron paramagnetic resonance (ESR/EPR) spectroscopy coupled to spin-trapping techniques provides direct and chemically specific detection of free radicals through the formation of stable paramagnetic adducts. Although this approach offers unmatched specificity, it requires specialized instrumentation, relatively high radical fluxes, and stringent experimental conditions, limiting its routine application in most laboratories.

Finally, all ROS measurements should be performed under physiologically relevant conditions whenever possible to minimize experimental artefacts. This consideration is particularly important in hypoxia studies, where reoxygenation during probe loading can substantially alter cellular redox status. Strategies such as probe incubation and fixation within hypoxia workstations [29,30], or live-cell imaging under controlled O_2_ conditions [31], help preserve physiological O_2_ tensions and improve the reliability of redox measurements.

### 2.3 Tools for quenching ROS

The tools available for ROS quenching encompass both the direct chemical removal of oxidants and the modulation of endogenous antioxidant defenses, and these two approaches must be clearly distinguished when antioxidants are used under experimental conditions. Acute ROS scavenging generally produces localized and immediate cellular effects, whereas long-term enhancement of antioxidant capacity may lead to markedly different biological outcomes, including the activation of distinct transcriptional programs and adaptive responses. Consequently, an appropriate interpretation of antioxidant-based experiments requires careful consideration of the quenching strategy employed, the specific ROS species targeted, the subcellular compartment involved, and the temporal scale of the intervention.

#### 2.3.1 Genetically encoded antioxidants

**Fig. 2:**
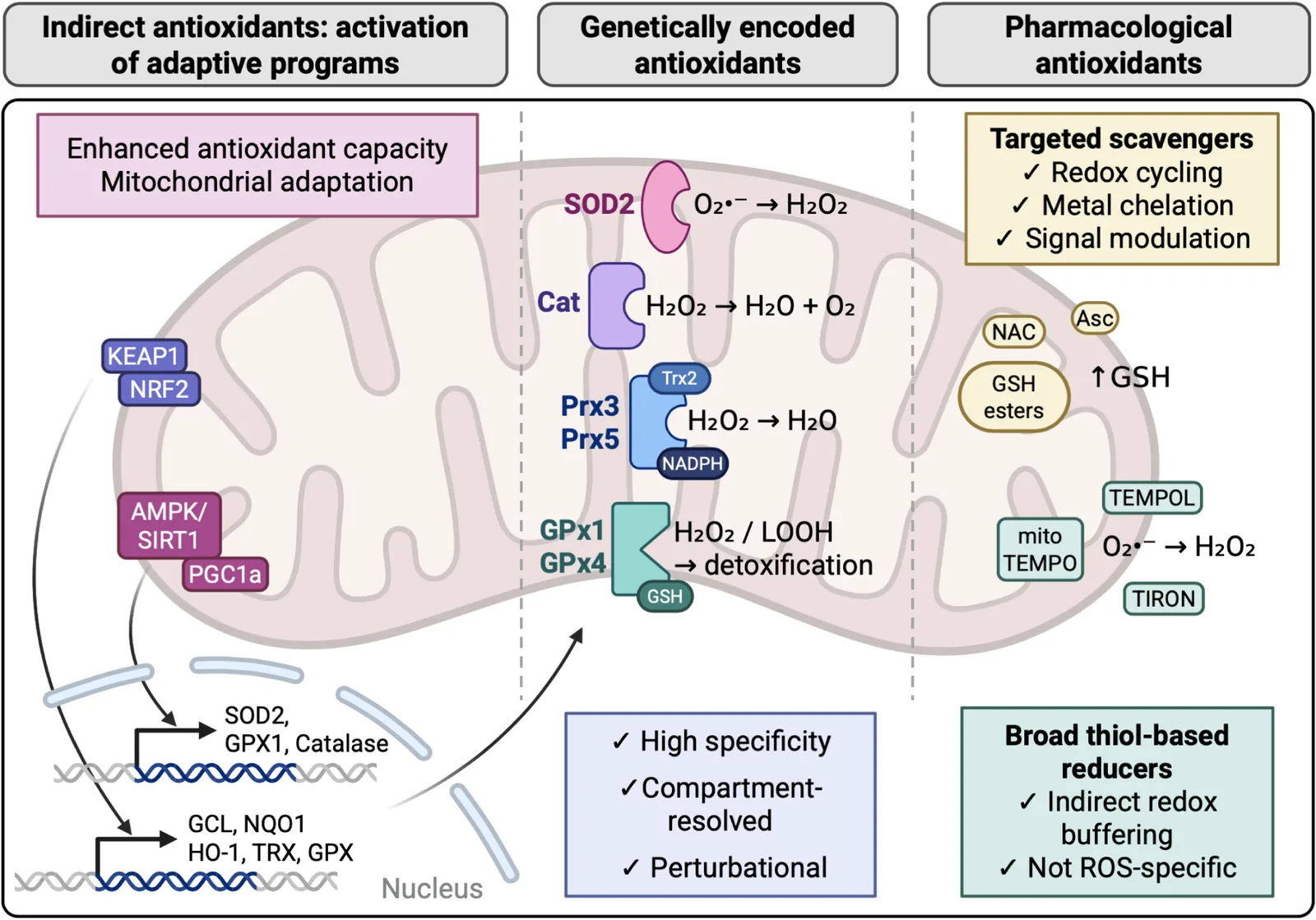
**Conceptual framework for ROS quenching strategies: direct scavenging versus induction of endogenous antioxidant defenses.** Direct ROS scavenging strategies are divided into genetically encoded and pharmacological antioxidants. Genetically encoded antioxidant enzymes, including catalases, PRXs, GPXs and SODs, allow compartment-resolved manipulation of specific ROS-detoxifying pathways, particularly within mitochondria, but inherently perturb endogenous redox networks and metabolic coupling. Only specific reactions, rather than the entire mitocohondrial antioxidant system, are depicted. Pharmacological antioxidants act either as broad thiol-based reducing agents (e.g., N-acetylcysteine, GSH esters and ascorbate), which indirectly modulate cellular redox buffering capacity, or as targeted scavengers (e.g., Tiron, TEMPOL and mito-TEMPO), which may undergo redox cycling, metal chelation or compartmental accumulation, complicating mechanistic interpretation. In contrast, indirect antioxidant strategies induce endogenous defense programs through activation of transcriptional regulators such as NRF2 or PGC-1α, leading to coordinated upregulation of antioxidant enzymes, redox cofactors and mitochondrial biogenesis over longer time scales.

Genetically encoded antioxidant enzymes represent some of the most precise tools available for studying ROS quenching mechanisms, as they enable the selective manipulation of specific detoxification pathways with high subcellular resolution. By enhancing or disrupting endogenous antioxidant systems, these approaches facilitate the functional identification of the ROS species, cellular compartments, and enzymatic pathways responsible for a given phenotype [32] (Fig. 2).

a) Catalase exemplifies the utility of compartment-specific genetic antioxidants. Although endogenous catalase expression within the mitochondrial matrix is low in most mammalian cells, engineered targeting of catalase to the cytosol, nucleus, or mitochondrial intermembrane space (IMS) provides a precise means of eliminating H_2_O_2_ locally. Consequently, catalase is an excellent tool for determining whether H_2_O_2_ is required for a specific biological response [33,34]. Differential compartmental expression of catalase can also help establish whether mtROS act locally or through diffusion-mediated redox signaling with other cellular compartments [35].
b) Mitochondrial PRX3 and PRX5 exhibit exceptionally high reactivity toward H_2_O_2_ and are functionally coupled to the TRX2/TRXR2/NADPH system, enabling the rapid and reversible regulation of local peroxide levels [36,37]. The accumulation of hyperoxidized PRXs provides a mechanistic threshold that distinguishes physiological redox signaling from oxidative stress [38]; Furthermore, targeted expression of PRX5 within the IMS has proven effective in quenching physiological redox signaling during hypoxia [39].
c) Genetic manipulation of GPXs provides valuable insight into the pathways involved in ROS detoxification under the physiological or pathological conditions being studied [40,41]. The functional redox state of mitochondrial GPXs, particularly GPX1 and GPX4, has frequently been used as an indirect indicator of oxidative burden [42,43]. Because GPX activity is strictly dependent on the availability of GSH, sustained mitochondrial oxidative stress can compromise GPX catalytic capacity through either GSH depletion or the accumulation of lipid hydroperoxides [41]. In this context, impaired GPX activity or increased sensitivity to ferroptotic stimuli may reflect a broader redox imbalance rather than isolated enzyme dysfunction.
d) Mitochondrial superoxide dismutase (SOD2) catalyzes the dismutation of matrix-derived O_2_•^-^ into H_2_O_2_, thereby allowing discrimination between processes driven by O_2_^•-^ itself and those mediated by downstream peroxide signaling [44,45]. SOD1 is localized to both the cytosol and the IMS, where it can quench O_2_•^-^ generated by the electron transport chain (ETC), particularly through specific ROS-producing modes involving complex III [46]. Increased SOD activity may shift the oxidative burden toward H_2_O_2_, thereby elevating peroxide levels and potentially enhancing H_2_O_2_-dependent signaling pathways.
e) Manipulation of GSH synthesis constitutes a particularly informative strategy for determining whether a biological phenotype depends on the size and availability of the intracellular GSH pool. Genetic modulation of glutamate–cysteine ligase (GCL), the rate-limiting enzyme in GSH biosynthesis, is especially useful. GCL consists of a catalytic subunit (GCLC) and a modifier subunit (GCLM), and altering the expression of either component enables controlled changes in total cellular GSH levels and, consequently, in the capacity of the cell to sustain GSH-dependent ROS detoxification [47]. Because GSH is required for GPX- and GRX-dependent antioxidant pathways, these manipulations can help determine whether the observed phenotype is specifically constrained by GSH availability rather than by ROS production alone.
f) Genetic manipulation of the mitochondrial TRX/PRX system represents one of the most informative approaches for investigating rapid H_2_O_2_ detoxification and peroxide-dependent signaling. This system, centered on TRX2, TRXR2, and PRX3—with contributions from PRX5 in certain contexts—constitutes a major high-affinity peroxide-buffering pathway within mitochondria and is particularly well suited to controlling rapid and localized fluctuations in H_2_O_2_. Because PRXs react with H_2_O_2_ with exceptionally high efficiency and are subsequently recycled by TRX2 at the expense of TRXR2 and NADPH, perturbation of any component of this pathway enables the investigator to determine whether a phenotype depends on this rapid thiol-based detoxification circuit rather than on GSH-dependent antioxidant mechanisms. Accordingly, the TRX/PRX axis functions not only as an antioxidant system but also as a key regulator of whether local peroxide signals are quenched, propagated, or allowed to accumulate [[48], [49], [50]].
g) Targeting γ-glutamylcysteine (γ-GC) metabolism provides a useful and relatively selective strategy for assessing the contribution of the mitochondrial GPX pathway to peroxide detoxification. γ-GC is the immediate precursor of GSH and can itself support peroxide removal in mitochondria through GPX1-dependent reactions. Consequently, mitochondrial generation of γ-GC through the expression of mitochondrial-targeted glutamate–cysteine ligase (mitoGCL), or supplementation with exogenous γ-GC when experimentally feasible, can be used as a functional tool to determine whether the phenotype under investigation depends on this branch of GSH-linked antioxidant defense. This approach is particularly informative because it can help distinguish GPX1-dependent peroxide buffering from alternative antioxidant pathways, such as the TRX/PRX system, or from broader changes in cellular redox homeostasis [25,51].

#### 2.3.2 Pharmacological antioxidants

Pharmacological antioxidants are also widely used to investigate ROS-quenching mechanisms and are often among the first tools employed to determine whether a given phenotype depends on oxidative processes. However, their effects must be interpreted with caution, as most compounds classified as antioxidants do not function as selective or straightforward ROS scavengers.

a) Broad thiol-based reducing agents, such as N-acetylcysteine (NAC), GSH esters, and ascorbate, primarily modulate intracellular redox buffering by replenishing or redistributing thiol pools rather than by directly neutralizing specific ROS [52]. For example, NAC increases cysteine availability for GSH synthesis, promotes the reduction of protein thiols [53], and indirectly enhances antioxidant defenses while simultaneously affecting redox-sensitive signaling pathways, mitochondrial metabolism, and gene expression. Consequently, reductions in ROS-related readouts following NAC treatment cannot be simply attributed to direct ROS scavenging but rather reflect a broader reprogramming of cellular redox homeostasis [54].
b) Targeted pharmacological scavengers are designed to neutralize specific ROS species or subcellular ROS pools; however, their selectivity is often incomplete and highly context dependent. Tiron has frequently been used as an O_2_^•-^ scavenger, although its metal-chelating properties and limited mitochondrial permeability complicate mechanistic interpretation [55]. Nitroxide radicals such as TEMPOL and the mitochondria-targeted derivative mito-TEMPO exhibit O_2_^•-^ and ONOO^−^ scavenging activity. The latter is commonly employed to assess the involvement of mtROS, but caution is warranted because its mitochondrial accumulation depends on ΔΨmt. Furthermore, these compounds can participate in redox cycling, alter ΔΨmt, and influence ETC activity, thereby indirectly affecting ROS production [56,57]. Similar considerations apply to positively charged benzoquinone derivatives such as SkQ compounds, which are structural analogs of coenzyme Q, plastoquinone, and tocopherol (vitamin E). Owing to their positive charge, these molecules accumulate within mitochondria, where reduced SkQ hydroquinones can act as free-radical scavengers. However, they may also exert antioxidant effects in non-mitochondrial compartments, complicating the interpretation of their selectivity toward mtROS signaling [58].
c) Lipid-soluble antioxidants, such as α-tocopherol, constitute another important class of pharmacological redox modulators, particularly in the context of membrane lipid peroxidation. Tocopherols primarily function as chain-breaking antioxidants by interrupting the propagation of lipid peroxyl radicals within biological membranes [59,60]. Although this activity can attenuate lipid peroxidation-driven damage, it does not directly affect upstream ROS generation and may selectively modulate membrane-associated oxidative processes without substantially altering cytosolic redox signaling [61].

Likewise, lipid-soluble mitochondria-targeted antioxidants such as MitoQ consist of redox-active quinones conjugated to lipophilic cations that accumulate within the mitochondrial matrix under the influence of ΔΨmt. Following reduction to ubiquinol, MitoQ can limit lipid peroxidation and modulate mitochondrial redox status [62]. However, because MitoQ is itself a redox-cycling molecule, it may also influence ETC activity and ROS production rates, thereby altering mitochondrial signaling beyond simple ROS scavenging [63]. Finally, in contrast to classical antioxidants, electrophilic lipid peroxidation products such as 4-hydroxynonenal (HNE) function as endogenous pro-oxidant signaling mediators [64]. HNE covalently modifies cysteine, histidine, and lysine residues within proteins, thereby altering enzyme activity and activating stress-response pathways, including Nuclear factor erythroid 2–related factor 2 (NRF2)-dependent signaling [65]. Although often regarded as markers of oxidative damage, these reactive lipid aldehydes also serve as bona fide redox signaling molecules, highlighting that lipid oxidation products can both propagate oxidative stress and initiate adaptive antioxidant responses, depending on their concentration and duration of exposure.

d) Enzyme inhibitors, such as TRX reductase (TRXR) inhibitors including auranofin, can provide valuable mechanistic information but frequently exhibit broad off-target effects. Consequently, they are generally best employed as supporting evidence rather than as the sole basis for mechanistic conclusions.

Taken together, pharmacological antioxidants are inherently perturbative interventions that can trigger compensatory metabolic adaptations and transcriptional responses. Chronic antioxidant exposure may alter mitochondrial metabolism, NADPH utilization, and redox signaling networks, thereby masking the primary mechanisms driven by ROS. Therefore, antioxidant-based interventions should always be interpreted with caution, as their intrinsic metabolic and signaling effects may confound the attribution of observed phenotypes to specific ROS-dependent processes.

#### 2.3.3 Activation of endogenous antioxidant transcriptional programs

A large class of compounds exerts its primary effects not through direct ROS scavenging, but rather by activating endogenous antioxidant defense systems. These effects typically involve redox-sensitive transcriptional and metabolic pathways that progressively enhance cellular ROS detoxification capacity rather than acutely reducing oxidant levels. The principal transcriptional programs involved include:

a) Activation of the NRF2 pathway. This represents one of the major mechanisms through which electrophilic or mildly pro-oxidant compounds stimulate the expression of genes involved in GSH synthesis, ROS detoxification, and redox recycling, including glutamate–cysteine ligase, glutathione reductase (GR), glutathione peroxidases (GPXs), PRXs, and SODs [66,67] (see Section 5.2 for a detailed discussion of this pathway).
b) Activation of Peroxisome proliferator-activated receptor γ coactivator-1α (PGC-1α). Activation of PGC-1α promotes the transcriptional upregulation of several mitochondrial antioxidant enzymes, including SOD2 and GPX1 [68], thereby enhancing mitochondrial resistance to oxidative stress (see Section 5.2 for further details regarding the function of this pathway).
c) Enhancement of reducing-equivalent availability. By increasing the availability of reducing equivalents, these adaptive programs support the activity of both TRX- and glutathione (GSH)-dependent antioxidant systems, thereby improving mitochondrial resilience to sustained oxidative stress (see Section 5.2 for additional details regarding these mechanisms).
d) Importantly, these adaptations can also alter mitochondrial content, respiratory capacity, and ROS production rates, thereby complicating the interpretation of their specific redox-dependent effects.

In conclusion, the selection of antioxidant tools requires careful conceptual understanding and rigorous experimental design. Both genetic and pharmacological antioxidants influence redox fluxes rather than simply reporting oxidative states, and their effects are strongly dependent on the ROS species involved, the subcellular compartment affected, the timing of the intervention, and the adaptive capacity of the cell. Consequently, antioxidant-based experiments should be interpreted within the broader context of cellular redox homeostasis rather than as direct measures of ROS scavenging alone.

## 3 Non-mtROS-producing enzymes

In this section, we describe several of the major ROS-producing enzymes located in non-mitochondrial compartments. Our aim is to provide a general overview of these enzymatic systems, which we have extensively studied over recent years, in order to familiarize the reader with the mechanisms that govern their activity and the factors that trigger and modulate ROS production outside the mitochondria. We believe that providing an overview of non-mtROS sources is essential to avoid an excessively mitochondria-centered perspective, which may lead readers to assume that mitochondria are the only, or invariably the main, source of ROS under all conditions.

Here, we will provide a detailed understanding of the biochemical mechanisms and spatial organization of these enzymes is essential for determining whether they contribute to specific redox events. Combined with the concepts introduced in Section 2, this knowledge will facilitate the design of appropriate experimental workflows for investigating these and other ROS-generating systems within the broader context of redox biology. In addition, this section will enable the reader to understand a common (patho)physiological mechanism of ROS amplification explained in Section 4.2.1.

This section is organized into four subsections: Section 3.1, NADPH oxidases (NOXs); Section 3.2, xanthine oxidoreductase (XOR); Section 3.3, peroxisomal enzymes; and Section 3.4, endoplasmic reticulum (ER) enzymes.

### 3.1 NADPH oxidases (NOXs)

**Fig. 3:**
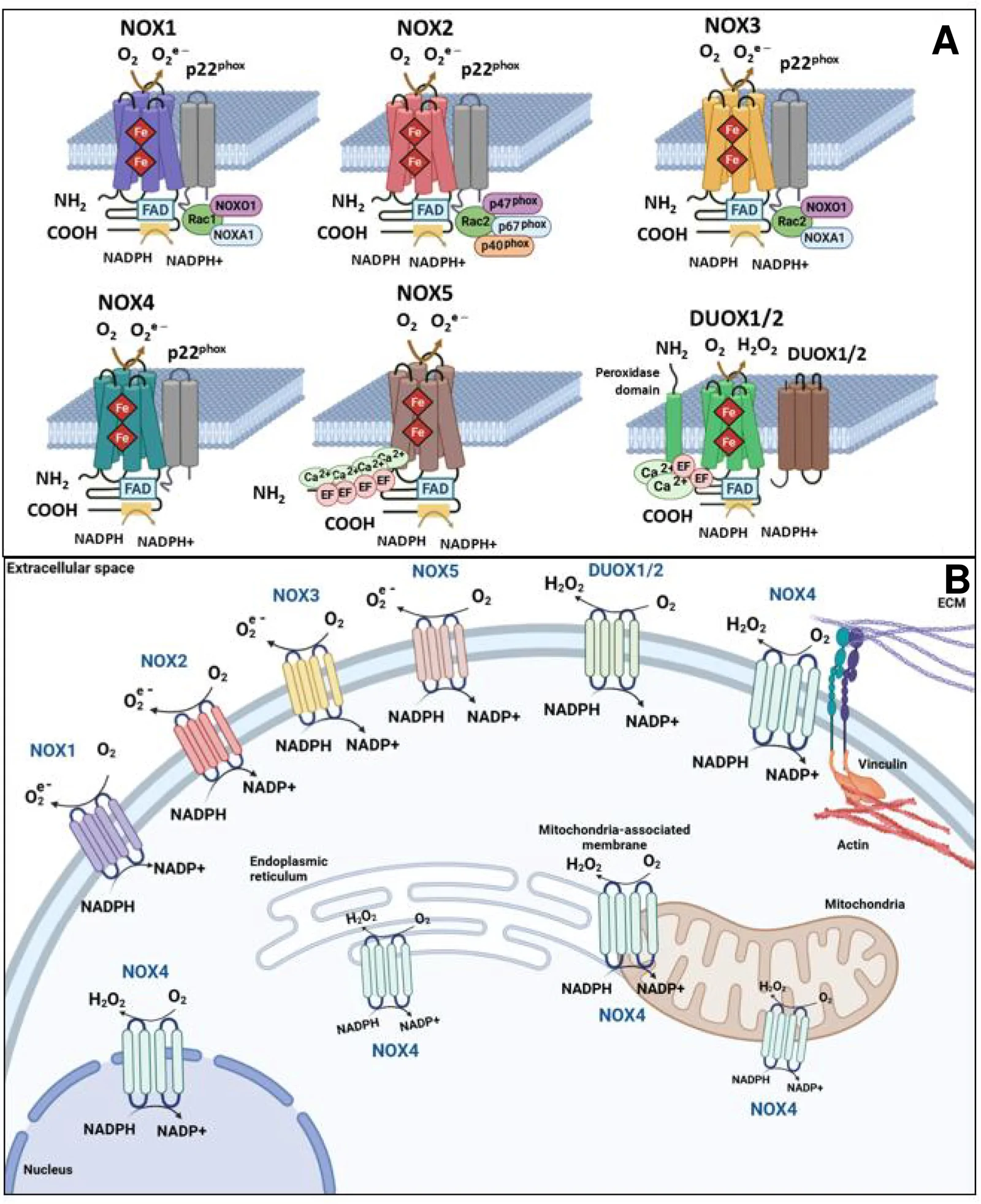
**Structural organization and localization of the NOX family.** A) Structural features and activation mechanisms of NOX family members. NOX1–3 require p22^phox^ and activation by cytosolic organizer and activator subunits together with Rac GTPases. NOX2 can use both Rac1 and Rac2. NOX4 displays constitutive activity and depends mainly on p22^phox^. NOX5 is activated directly by intracellular Ca^2+^ through EF-hand domains. DUOX1 and DUOX2 contain EF-hand and extracellular peroxidase-like domains and require DUOXA1 and DUOXA2 for maturation and functional expression. All NOX enzymes transfer electrons from NADPH to O_2_, generating O_2_•^-^ and/or H_2_O_2_. B) Representative subcellular localization of NOX isoforms. NOX1, NOX2, NOX3, NOX5, and DUOX1/2 are primarily located at the plasma membrane, whereas NOX4 is also found in the nucleus, ER, mitochondria-associated membranes (MAMs), and mitochondria. Differences in localization, regulation, and ROS output enable compartment-specific redox signaling and underlie the diverse physiological and pathological functions of NOX enzymes. See text for further details.

NOXs constitute a family of membrane-associated enzymes specialized in the controlled generation of ROS, thereby contributing to signaling processes in both physiology and pathology [69]. A defining characteristic of this enzyme family is the diversity of regulatory mechanisms, activation modes, and biological functions among its members, allowing finely tuned and context-dependent redox signaling. The mammalian NOX family comprises NOX1–5 and the dual oxidases DUOX1 and DUOX2, which differ substantially in their activation mechanisms, regulatory requirements, and ROS outputs [69] (Fig. 3A). In the following sections, we summarize the activation mechanisms and biological functions of the major NOX family members:

a) NOX1, NOX2, and NOX3, which are primarily localized to the plasma membrane (Fig. 3B), are activated through the stimulus-dependent assembly of cytosolic organizer and activator subunits, frequently in conjunction with Rac GTPases (Fig. 3A, NOX1-3 panels) [70]. This multicomponent regulatory system enables rapid and tightly controlled ROS production in response to extracellular stimuli, supporting functions in host defense, epithelial signaling, and sensory physiology. In particular, NOX2 is responsible for the phagocytic oxidative burst [71], whereas NOX1 and NOX3 are predominantly involved in epithelial signaling [72] and sensory functions [73], respectively.
b) NOX4, in contrast, exhibits a distinct regulatory paradigm characterized by high basal activity and limited dependence on classical cytosolic regulatory subunits (Fig. 3D, NOX4 panel). Its activity is regulated primarily at the transcriptional and post-transcriptional levels, and it predominantly generates H_2_O_2_ rather than O_2_^•-^ [74]. Together with its preferential localization to intracellular membranes (Fig. 3B), these properties support a role for NOX4 in sustained and compartmentalized redox signaling associated with cellular differentiation, metabolic regulation, and stress responses [75]. Transforming growth factor-β (TGF-β) is a potent inducer of NOX4 expression, acting mainly through SMAD-dependent transcriptional programs [76] that couple redox signaling to the diverse biological functions mediated by NOX4 [77].
c) NOX5 (localized to the plasma membrane and ER; Fig. 3B) and the dual oxidases DUOX1 and DUOX2 (localized primarily to the plasma membrane; Fig. 3B) constitute a third regulatory class of NOX enzymes, being directly activated by intracellular Ca^2+^ through EF-hand domains (Fig. 3A, NOX5 and DUOX1/2 panels) [78]. This mechanism enables the rapid coupling of ROS production to Ca^2+^ signaling pathways independently of organizer subunits [79]. The maturation of DUOX proteins depends on DUOXA1 and DUOXA2 (DUOX maturation factors), which function as molecular chaperones required for the proper folding, glycosylation, trafficking, and functional expression of DUOX1 and DUOX2, respectively (Fig. 3A, DUOX1/2 panels) [80]. DUOX enzymes additionally contain extracellular peroxidase-like domains and perform specialized functions in epithelial defense and hormone biosynthesis, particularly within the thyroid gland [81]. The physiological role of NOX5 remains difficult to evaluate in commonly used rodent models because this isoform is absent from both mice and rats [82], complicating *in vivo* studies and translational interpretation. NOX enzymes generate ROS by transferring electrons from cytosolic NADPH across biological membranes to molecular O_2_. This one-electron reduction primarily produces O_2_^•-^, which can subsequently dismutate into H_2_O_2_ [83]. The precise ROS output depends on the NOX isoform and cellular context. DUOX enzymes preferentially generate H_2_O_2_ due to the presence of their extracellular peroxidase-like domains. Although the initial electron-transfer reaction is similar to that of other NOX family members, DUOX proteins may rapidly convert the generated O_2_•^-^ into H_2_O_2_ before its release, making them efficient and relatively direct sources of extracellular H_2_O_2_ for signaling and host defense. Interestingly, specific domains of DUOX maturation factors influence not only DUOX activation but also the type of ROS produced. Native DUOXA2 constrains DUOX2 to generate H_2_O_2_, whereas alterations in the amino-terminal domain of DUOXA2 promote O_2_^•-^ leakage from DUOX2 [84].

Functionally, the diversity in regulation and subcellular localization among NOX isoforms gives rise to distinct ROS-signaling outputs that influence specific cellular pathways [69]. These differences are central to understanding how NOX enzymes mediate spatially and temporally controlled redox signaling under physiological conditions [69], contribute to protective functions in human disease [85,86] and participate in pathological processes when dysregulated, including chronic inflammation [87,88], fibrosis [89,90], cancer progression [77,91], cardiovascular diseases [92,93], or neurological disorders [94,95].

An additional unresolved issue in the NOX field concerns subcellular localization and compartmentalization [96]. Depending on the experimental system and detection methodology employed, individual NOX isoforms have been reported at the plasma membrane, ER, mitochondria-associated membranes, nucleus, and endosomal compartments (Fig. 3B). This variability has important implications for the interpretation of NOX-dependent signaling specificity and highlights the need for improved tools capable of assessing isoform-specific activity *in situ* [96,97].

Importantly, NOX function is intimately linked to mitochondrial activity and mtROS production. Early studies demonstrated a bidirectional crosstalk in which mtROS promote NOX-dependent oxidative stress, whereas NOX-derived ROS amplify endothelial injury [98]. These findings suggested that mitochondria often initiate ROS production, whereas NOX enzymes serve as amplifiers, with major consequences for vascular biology. Subsequent work demonstrated that mtROS activate NOX2 in phagocytes and cardiovascular tissues, promoting endothelial dysfunction [99]. These observations revealed a key concept: NOX enzymes can act as amplification platforms for mtROS signaling, thereby contributing to pathological outcomes. This phenomenon has been described in feed-forward signaling pathways involving NLRP3 inflammasome activation, in which oxidative stress promotes dissociation of TXNIP from TRX and facilitates its interaction with NLRP3 [85,97]. Consequently, ROS amplification between mitochondria and NOX enzymes is bidirectional [100], and has been implicated in conditions such as hypoxia, angiotensin II-induced hypertension, nitrate tolerance, and aging [101].

Conversely, mitochondrial dysfunction can also arise downstream of NOX activation. For example, angiotensin II-induced activation of NOX enzymes promotes mitochondrial damage, which subsequently decreases nitric oxide bioavailability and further enhances oxidative stress, identifying mitochondria as both targets and amplifiers of angiotensin II-driven endothelial dysfunction [102]. Furthermore, NOX2 plays an essential role in maintaining mitochondrial respiration and ATP production in hematopoietic stem cells by sustaining redox-dependent signaling pathways required for respiratory chain function. Accordingly, NOX2 deficiency impairs ETC activity, decreases ATP production, and disrupts stem-cell homeostasis through alterations in mitochondrial metabolism and mtROS signaling [103].

In contrast, serum withdrawal induces an mtROS burst that initiates a signaling cascade leading to NOX1 activation through the PI3K–Rac1 pathway. In this setting, mtROS activate NOX1, which subsequently generates a second and sustained wave of ROS that maintains oxidative stress and ultimately promotes cell death. This feed-forward mechanism, known as ROS-induced ROS release (RIRR), exemplifies how mitochondria and NOX enzymes cooperate to regulate ROS production and generate oxidant signals appropriate to cellular demands [104].

### 3.2 Xanthine oxidoreductase (XOR)

XOR is a cytosolic enzyme encoded by the human xanthine dehydrogenase (XDH) gene. XOR is initially synthesized as an NAD^+^-dependent xanthine dehydrogenase (XDH), which can subsequently be converted into xanthine oxidase (XO) through intermediate forms, particularly following release into the extracellular milieu [105]. XDH, XO, and their intermediate forms catalyze two sequential hydroxylation reactions: the conversion of hypoxanthine to xanthine and of xanthine to uric acid (UA). Electrons released during these reactions are transferred either to NAD ^+^ or to molecular O_2_, depending on the enzymatic state. In its dehydrogenase form (XDH), NAD^+^ gains access to the FAD domain and is reduced to NADH, although XDH may also utilize O_2_ as an electron acceptor when NAD ^+^ availability is limiting. In contrast, the oxidase form (XO) prevents NAD ^+^ binding to the FAD domain and instead permits O_2_ access to the solvent channel102. Under these conditions, the enzyme reduces O_2_ to either O_2_•^-^ or H_2_O_2_ through one- or two-electron reduction reactions, respectively.

XOR is ubiquitously expressed [106], although its highest expression and activity are found in the liver, small intestine, and lactating mammary glands [107]. In pathological conditions, including cardiovascular and metabolic diseases, circulating XOR levels increase markedly. This increase is accompanied by substantial release of XDH into the bloodstream, where it is progressively converted into the oxidase form [106]. In addition, nonsynonymous mutations in the human XDH gene can influence both pre- and post-translational regulation, affecting not only enzyme expression but also total XOR abundance and the relative proportion of XDH and XO isoforms [108].

XOR activity is closely linked to mitochondrial function. In models of acute cardiac volume overload, increased XOR activity coincides with reduced subsarcolemmal mitochondrial state 3 respiration and mitochondrial swelling. Importantly, treatment with allopurinol, a classical XOR inhibitor, prevents or normalizes these mitochondrial alterations and improves early hemodynamic dysfunction when administered at the onset of overload. These findings suggest that early XOR–mitochondria crosstalk may contribute to the progression from volume overload to heart failure [109].

Furthermore, XOR and the ETC can cooperate to sustain vascular oxidative stress in mineralocorticoid-induced hypertension. In salt-induced hypertensive rats, vascular O_2_•^-^ production originates not only from NOX enzymes but also, to a substantial extent, from XOR and mitochondrial ETC-derived sources. Pharmacological inhibition of XOR with allopurinol, blockade of endothelin receptors using bosentan or the ETA-selective antagonist BMS-182874, and inhibition or uncoupling of mitochondrial respiration using the complex II inhibitor TTFA or the uncoupler CCCP—but notably not the complex I inhibitor rotenone—significantly reduce O_2_•^-^ production in mesenteric resistance arteries and the aorta [110]. These observations highlight the functional interplay between XOR activity and mtROS generation in the maintenance of vascular oxidative stress.

Interestingly, XOR has been detected in both cytosolic and mitochondrial compartments during inflammasome activation [111], a finding that may stimulate further investigation into its compartment-specific roles and its contribution to mitochondrial redox signaling.

### 3.3 Generation of ROS by peroxisomal enzymes

Peroxisomes are highly dynamic, single-membrane-bound eukaryotic organelles that adjust their abundance, morphology, and metabolic activity according to tissue type, organ function, and nutritional status [112]. In mammals, peroxisomes participate in numerous metabolic pathways, including fatty acid α- and β-oxidation, ether phospholipid biosynthesis, bile acid and docosahexaenoic acid synthesis, glyoxylate metabolism, amino acid catabolism, and polyamine oxidation [112]. They are also major intracellular sources of H_2_O_2_, which is generated as a byproduct of oxidative metabolism primarily through the action of oxidases [113], particularly flavin-containing oxidases. The principal peroxisomal oxidases and the pathways in which they participate are summarized below.

a) Acyl-CoA oxidases (ACOXs) catalyze the rate-limiting first step of the peroxisomal β-oxidation pathway, which shortens long-chain fatty acids, branched-chain fatty acids, and bile acid intermediates. These enzymes transfer electrons from acyl-CoA substrates to molecular O_2_, generating H_2_O_2_ as a byproduct while utilizing FAD as a cofactor. Interestingly, the interaction between ACOX enzymes and FAD is allosterically regulated by ATP, providing a link between cellular energy status and lipid metabolism [114]. Three ACOX isoforms (ACOX1–3) have been identified, each exhibiting distinct substrate specificities, and their dysfunction is associated with severe inherited disorders.

ACOX1 primarily oxidizes straight-chain fatty acyl-CoAs. Two isoforms have been described: isoform 1 displays the highest activity toward medium-chain fatty acyl-CoAs, whereas isoform 2 exhibits a broader substrate spectrum with marked activity toward long-chain fatty acyl-CoAs [115]. Homozygous mutations causing ACOX1 deficiency result in pseudoneonatal adrenoleukodystrophy, a severe neurological disorder characterized by hypotonia, seizures, progressive blindness and deafness, increased muscle tone, and accumulation of very-long-chain fatty acids [116]. In contrast, Mitchell syndrome arises from heterozygous gain-of-function mutations in ACOX1 that increase ROS production without affecting very-long-chain fatty acid metabolism. Clinically, this syndrome is characterized by episodic demyelination, sensorimotor polyneuropathy, and hearing loss [117].

ACOX2 oxidizes 2-methyl-branched fatty acids, very-long-chain fatty acids, and bile acid precursor esters [115]. The homozygous R225W missense mutation causes ACOX2 deficiency, a disorder characterized by accumulation of toxic C27 bile acid intermediates and persistent hypertransaminasemia [118]. ACOX3 preferentially oxidizes 2-methyl-branched fatty acids, long-chain fatty acids, and very-long-chain fatty acids [112,119]. Missense mutations such as F79V and G222E result in near-complete loss of ACOX3 activity and have been associated with vascular dysfunction, particularly recurrent vasospasm [120].

b) d-Amino acid oxidase (DAO) and d-aspartate oxidase (DDO) catalyze the oxidative deamination of amino acids, generating α-keto acids, NH_4_^+^, and H_2_O_2_ while utilizing FAD as a cofactor. Through the regulation of d-serine and d-aspartate concentrations—both endogenous modulators of NMDA receptor activity—these enzymes influence neurotransmission and brain function. Altered levels of these amino acids have been associated with several neurological disorders [121].
c) 2-Hydroxyacid oxidases (HAOs) catalyze the oxidation of 2-hydroxy fatty acids, producing H_2_O_2_ as a byproduct while utilizing FMN as a cofactor. These enzymes play essential roles in glyoxylate metabolism, fatty acid α-oxidation, and redox signaling. Three isoenzymes (HAO1–3) have been identified, each displaying distinct tissue-specific expression patterns and substrate preferences [122].

HAO1 is expressed predominantly in the liver and pancreas, with lower levels detected in the kidney. Its preferred substrate is glycolate, which is oxidized to glyoxylate, although it can also metabolize 2-hydroxy fatty acids. HAO2 is primarily expressed in the liver and kidney and exhibits the highest activity toward long-chain 2-hydroxy acids, particularly 2-hydroxypalmitate. HAO3 expression has been reported exclusively in the pancreas and shows a preference for the medium-chain substrate 2-hydroxyoctanoate. Several homozygous mutations causing HAO1 deficiency have been linked to hyperoxaluria and renal stone disease [123].

d) Peroxisomal polyamine oxidase (PAOX) is a key enzyme in polyamine metabolism. It catalyzes the oxidation of N-acetylspermine to spermidine and N-acetylspermidine to putrescine, generating H_2_O_2_ as a byproduct while utilizing FAD as a cofactor [124]. In mammals, alternative splicing of the PAOX gene produces isoforms with distinct biochemical properties and functions in polyamine catabolism [125] By regulating intracellular polyamine levels, PAOX prevents the excessive accumulation of higher-order polyamines while linking polyamine turnover to broader cellular processes, including redox homeostasis and stress responses [125]. Because cancer cells and other pro-tumorigenic cell populations often depend on elevated polyamine pools for survival and proliferation, pharmacological targeting of polyamine metabolism has emerged as a promising therapeutic strategy [126].
e) Peroxisomes contain not only ROS-producing oxidases but also an extensive repertoire of antioxidant enzymes. One of the first antioxidant enzymes ever described was identified together with H_2_O_2_ itself in 1818 [127] and was later named catalase by Oscar Loew in 1900 [128]. Catalase remains one of the most abundant antioxidant enzymes within peroxisomes and serves as the principal mechanism for eliminating excess H_2_O_2_. In addition, peroxisomes contain SOD1, PRX5, GSH S-transferase kappa (GSTK1), and substantial pools of GSH, all of which contribute to their redox-buffering capacity. These properties establish peroxisomes as critical hubs of cellular redox regulation. To perform their physiological functions, peroxisomes must engage in both functional and physical interactions with other organelles, particularly mitochondria. These organelles cooperate in numerous metabolic and signaling pathways, and dysfunction in one frequently affects the other [129]. Such defects can result in progressive organelle dysfunction, increased ROS production, and activation of pexophagy, the selective autophagic degradation of peroxisomes [130].

More recently, an important role for peroxisomes in mitochondrial redox homeostasis has been uncovered. Peroxisomes actively contribute to the maintenance of mitochondrial redox balance by functioning as an extramitochondrial antioxidant compartment. In particular, a peroxisome–mitochondria contact site formed by Acbd5 and Ptpip51 increases in abundance during mitochondrial oxidative stress. This interaction facilitates the transfer and detoxification of mtROS within the peroxisomal lumen, thereby limiting the accumulation of oxidative products within mitochondria and promoting mitochondrial fitness. These findings demonstrate that membrane contact sites between mitochondria and peroxisomes can directly participate in mtROS handling between organelles, expanding the role of peroxisomes beyond metabolism and establishing them as contact-dependent redox “overflow” systems for mitochondria [131].

### 3.4 Generation of ROS by endoplasmic reticulum (ER) enzymes

The ER maintains a high turnover of H_2_O_2_, as its oxidative protein-folding machinery continuously generates and consumes this molecule. The core of the ER oxidative-folding system consists of a group of chaperone oxidoreductases belonging to the protein disulfide isomerase (PDI) family [132]. During oxidative protein folding, H_2_O_2_ is generated as a byproduct and contributes to the reoxidation of PDI through reactions involving PRX4 [133]. ER-derived H_2_O_2_ exerts both signaling and pathological effects, influencing cellular homeostasis [134] and even organismal longevity [135].

NOX enzymes and XOR represent major ROS-generating systems that interact closely with ER stress and mitochondrial dysfunction to promote cellular damage and disease. Both NOXs and XOR are frequently upregulated during ER stress, creating a vicious cycle in which ER-derived ROS, NOX-derived ROS, and mtROS mutually reinforce one another, leading to enhanced Ca^2+^ signaling, protein misfolding, and ultimately apoptosis [136]. ER stress activates the unfolded protein response (UPR), which can further increase ROS production through ERO1α activity, thereby promoting mitochondrial dysfunction and additional activation of NOX- and XOR-dependent ROS production [137].

The ER also maintains a particularly close functional and physical relationship with mitochondria. At mitochondria-associated membranes (MAMs), the protein kinase RNA-like ER kinase (PERK) functions not only as a stress sensor but also as a structural tether that facilitates Ca^2+^ and ROS signaling between the ER and mitochondria. Consequently, loss of PERK reduces MAM formation, diminishes ER-to-mitochondria Ca^2+^ and ROS transfer, and attenuates ROS-dependent apoptosis [138]. Importantly, the ER–mitochondria axis can also establish a self-amplifying ROS circuit. Pathogen-induced mtROS production can trigger ER stress, leading to the release of Ca^2+^ from the ER toward mitochondria. Increased mitochondrial Ca^2+^ uptake subsequently enhances mtROS generation and mitochondrial damage. Elevated mtROS, in turn, further stimulate ER Ca^2+^ release, establishing a positive feedback loop that amplifies oxidative stress [139].

It should also be noted that additional cytosolic enzymes can be modulated by mtROS. One prominent example is endothelial nitric oxide synthase (eNOS), whose activity is highly sensitive to changes in cellular redox state. mtROS can promote eNOS uncoupling—that is, the transition from nitric oxide (NO) production to O_2_^•-^ generation—through several redox-dependent mechanisms, including oxidation of the cofactor tetrahydrobiopterin (BH_4_), S-glutathionylation, and redox-sensitive phosphorylation events. As a result, eNOS is converted from an NO-producing enzyme into a source of O_2_^•-^, thereby exacerbating endothelial dysfunction and oxidative stress [140].

## 4 mtROS production, physiology and disease

Mitochondria are double-membrane organelles composed of an outer mitochondrial membrane (OMM) and an inner mitochondrial membrane (IMM), separated by the intermembrane space (IMS), which surrounds the mitochondrial matrix. Reduced cofactors, such as nicotinamide adenine dinucleotide (NADH), donate electrons to the mitochondrial ETC. NADH diffuses to and serves as a substrate for complex I (CI), which transfers electrons to ubiquinone (CoQ), reducing it to ubiquinol (CoQH_2_). In parallel, several FADH_2_-dependent enzymes transfer electrons from a variety of substrates to CoQ, thereby generating CoQH_2_; the most prominent example is complex II (CII). Complex III (CIII) oxidizes CoQH_2_ and reduces cytochrome *c* (cyt *c*), which subsequently transfers electrons to complex IV (CIV), where molecular O_2_ is reduced to water.

Complexes I, III, and IV couple electron transfer to proton (H^+^) translocation across the IMM into the IMS, generating a chemical H^+^ gradient (higher H^+^ concentration in the IMS and a more alkaline matrix), commonly referred to as ΔpH. The increased proton concentration within the IMS activates the Na^+^/H^+^ antiporter activity of CI, leading to the establishment of a mitochondrial Na^+^ gradient [141]. Together, these ion gradients generate the mitochondrial membrane potential (ΔΨmt), which, in combination with ΔpH, constitutes the proton-motive force (Δp) [141]. It is also important to note that CI, CIII, and CIV can assemble into higher-order quaternary structures known as supercomplexes.

Δp drives H^+^ re-entry into the matrix through ATP synthase, or complex V (CV), a rotary nanomachine that couples H^+^ translocation to the phosphorylation of adenosine 5′-diphosphate (ADP), generating adenosine 5′-triphosphate (ATP). When O_2_ consumption is *coupled* to ADP phosphorylation, mitochondrial respiration is said to be coupled to ATP synthesis. Complexes I–IV collectively constitute the mitochondrial ETC, which, together with ATP synthase, forms the oxidative phosphorylation (OxPhos) system.

In this section, we first review the mechanisms by which mitochondria generate ROS. Understanding the distinct pathways responsible for mtROS production is essential for identifying specific molecular targets that can be manipulated when a particular ROS source is suspected within an experimental workflow. We then present several case studies illustrating the physiological roles of mtROS signaling, thereby highlighting the biological relevance of specific mechanisms of mtROS generation. Finally, we examine how pathological increases in mtROS, when exceeding the capacity of antioxidant buffering systems, lead to oxidative damage and establish feed-forward cycles of bioenergetic and metabolic dysfunction. Particular emphasis is placed on pathological contexts that have been the focus of our work over recent years.

Overall, this section is intended to provide the reader with a mechanistic understanding of how mtROS are generated and why specific modes of ROS production are relevant in both physiological and pathological settings. Together with the information in sections 2, 3, this section provides the mechanistical and conceptual framework necessary for the design of robust and informative experimental workflows in mitochondrial redox biology. It is to note that the knowledge on the mitochondrial antioxidant systems (Section 5) is mandatory to completely and successfully generate a workflow.

### 4.1 Mechanisms of mtROS production

mtROS production varies dramatically according to bioenergetic state, substrate availability, and O_2_ tension, including differences between NADH- and CoQH_2_-dependent metabolism and between physioxic and normoxic conditions [142]. Although CI and CIII are prominent sources of O_2_^•-^ in many systems during forward (i.e., physiological) electron transfer [143], up to twelve mitochondrial enzymes and pathways have been identified as potential sources of ROS within this organelle. In general terms, the bioenergetic state profoundly influences mtROS production. The addition of NADH-generating substrates and/or succinate alone tends to promote mtROS generation, whereas their co-application with ADP reduces it [144]. In the following sections, we summarize the enzymes currently known to produce mtROS:

a) During coupled respiration supported by glutamate/malate or pyruvate/malate, tricarboxylic acid (TCA) cycle dehydrogenases, such as OGDH, malate dehydrogenase (MDH) and PDH produce NADH that fuels CI. Under these conditions, electron transfer proceeds efficiently through the ETC to CIII and CIV, and O_2_^•-^ formation remains low but detectable, arising mainly from CI, CIII, and OGDH during forward electron transfer (FET). Table 2 shows the molecules enhancing and inhibiting this mechanism.

**Table 2:** Modes of mtROS production, including their substrates, inductors and blockers.

Origin	Substrates	Inductor	Blocker
FET (forward electron transport)	- CI and CIII: Pyruvate, malate and glutamate. - CIII: Succinate.	- CI: Rotenone, Piericidin A. - CIII: Antimycin A	- CI: DPI. - CIII: myxothiazol, stigmatellin or mucidin.
OGDH	2-oxoglutarate	- High matrix NADH/NAD^+^ ratio - Oligomycin + ADP	- Succinyl phosphonate (alamethicin permeabilized) - 3-methyl-2-oxopentanoate - 2-oxoadipate
PDH	Pyruvate	- Dichloroacetate + CaCl_2_ - High matrix NAD(P)H - Malate - Carnitine - 2-oxoadipate	- Acetyl-CoA
BCKDH	- 3-methyl 2-oxobutanoate - 3-methyl 2-oxopentanoate - 4-methyl 2-oxobutanoate	- Low matrix NADH - 2-oxoadipate	- Oligomycin + ADP - High matrix NADH
OADH	2-oxoadipate	- High matrix NAD(P)H	- ATP
CI RET (reverse electron transport)	- From CII: Succinate. - From GPDH: G3P	- High succinate/G3P - CV inhibitors or ATP	- CI and CII inhibitors (DPI, rotenone and piericidin A) - OxPhos uncouplers (FCCP, CCCP, etc)
GPDH	G3P	-	-
DHODH	Dihydroorotate	-	- Brequinar - Leflunomide
ETF:CoQ	- Octanoyl-carnitine - Palmitoyl-carnitine	- High NAD(P)H	-
CII-FET	Low succinate	-	Malonate
CII-RET	High succinate	-	Atpenin A5 and malonate
SET	- From CI: glutamate/malate - From CII-succinate (whole cells)	Monensin, Nigericin, FCCP (in normoxic cells)	Rotenone, piericidin A, myxothiazol, malonate, NCLX inhibitors
MAOs	Monoamines	-	- MAO A: Clorgyline, Moclobemide - MAO B: Rasagiline, Safinamide
b) Mitochondrial 2-oxoglutarate dehydrogenase complex (OGDH), Pyruvate dehydrogenase (PDH), and branched-chain 2-oxoacid dehydrogenase (BCKDH) are major matrix NADH-consuming sources of O_2_^•-^. Mechanistically, these complexes generate ROS primarily when they are oxidizing their respective substrates (2-oxoglutarate, pyruvate or branched-chain 2-oxoacid), and ROS emission rises as the availability of NAD^+^ becomes limiting (i.e., when the NADH/NAD^+^ ratio increases), which favors electron leak to O_2_ from redox-active centers within the complexes [145,146]. Notably, the production of O_2_^•-^ and H_2_O_2_ by OGDH can be modulated by increasing S-glutathionylation, whereas GRX2 system reverses S-glutathionylation, restoring OGDH activity and lowering O_2_^•-^ production [147] (Table 2).
c) Mitochondrial 2-oxoadipate dehydrogenase (OADH) complex produces O_2_^•-^ during oxidation of 2-oxoadipate, an intermediate of lysine and tryptophan catabolism [148] (Table 2).
d) When reducing equivalents are supplied predominantly through FAD-dependent pathways, most notably via succinate oxidation at CII, CoQ becomes highly reduced and, if mitochondria are hyperpolarized electrons can flow backwards from CoQH_2_ into CI. This reverse electron transfer (RET) reduces NAD^+^ and generates O_2_^•-^ at high rates, representing one of the most prominent modes of mtROS production observed in both physiological and pathological settings (Fig. 4A). Two important points that normally pass unnoticed are that: 1) the precise RET-associated ROS site within CI remains debated [[149], [150], [151]] and that 2) mitochondrial polarization is a pre-requisite to this type of mechanism (aka, the use of depolarizing agents, such as FCCP or ADP, are an essential marker to identify this mechanism; Table 2).

**Fig. 4:**
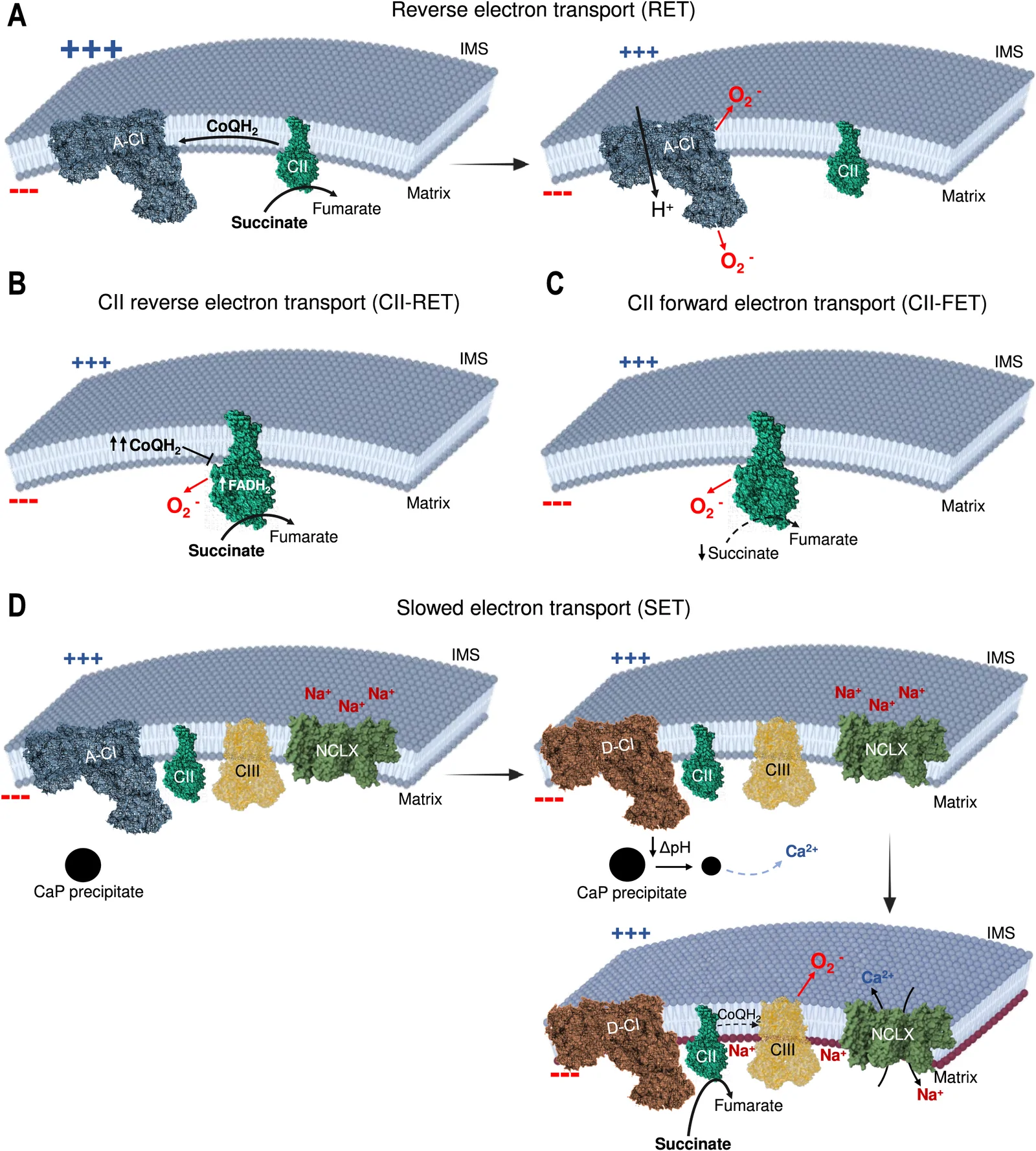
**Modes of mtROS production ny ETC complexes.** (A) RET by CI is produced by CoQH2 overproduction by FAD-containing enzymes, such as CII which, together with a hyperpolarized IMM produce the reverse reaction of active CI (A-CI). The specific site of O_2_^•-^ production is debated and both are depicted in scheme. (B) CII can generate O_2_^•-^ in a reverse mode after CoQH2 accumulation or FAD overreduction. (C) CII can also generate O_2_^•-^ in a forward mode when there is excesive succinate oxidation. (D) CIII can generate O_2_^•-^ after the accumulation of Na^+^ in the matrix. Normally, deactivation of CI (D-CI) or impeded mNHE activity precedes matrix Na^+^ accumulation.
e) Glycerol-3-phosphate (G3P) dehydrogenase (GPDH) is a major ROS source linked to cytosolic redox metabolism. This enzyme generates O_2_^•-^ primarily at its CoQ site during oxidation of glycerol-3-phosphate. Increasing G3P enhances O_2_^•−^production, whereas conditions that facilitate downstream electron flow to CoQ suppress it [152] (Table 2).
f) Mitochondrial dihydroorotate dehydrogenase (DHODH) produces O_2_^•-^ during its normal catalytic oxidation of dihydroorotate to orotate, in which electrons are transferred from substrate to the FMN cofactor and then to CoQ. When electron transfer rate from reduced FMN to CoQ is decreased or the CoQ pool is exceedingly reduced, partially reduced flavin intermediates can donate single electrons directly to O_2_, generating low but measurable amounts of ROS [153] (Table 2).
g) Electron-transferring flavoprotein:CoQ oxidoreductase (ETF:CoQ) generates O_2_^•-^ and H_2_O_2_ as a side reaction of its normal forward electron transfer from reduced ETF to CoQ. When electron supply exceeds CoQ oxidation or the CoQ pool is highly reduced, the ETF:CoQ flavin accumulates in reduced states that can transfer single electrons directly to O_2_, producing ROS [[154], [155], [156]] (Table 2).
h) Under specific conditions CII itself can become a significant mtROS source, with the flavin center as the principal emission site. In the normal forward reaction, succinate donates two electrons to FAD to form FADH_2_, which are passed to CoQ. However, FADH_2_ overreduction by either limited electron transfer to CoQ or by CoQH_2_ accumulation after CIII inhibition, translate into the transfer of single electrons directly to O_2_, forming O_2_^•-^ in the so-called CII-RET (Fig. 4B). In contrast to this, CII-derived O_2_^•-^ can also arise during forward succinate oxidation (CII-FET; Fig. 4C), under low succinate conditions [157] (Table 2).
i) There is a mechanism of mtROS production that increases O_2_^•-^ production involving two complexes. Deactivation of CI [158], which acidifies the matrix, mobilizes Ca^2+^ from the Ca^2+^ Pi precipitates (CaP precipitates) stores, and activates the mitochondrial Na^+^/Ca^2+^ exchanger (NCLX). The resulting Na^+^ influx translates into the accumulation of matrix Na^+^, which interacts with phospholipids in the inner leaflet of the IMM. This interaction produces 3phospholipid:1Na ^+^ adducts that decrease IMM fluidity and promotes O_2_^•-^ formation at the outer site of CIII, or Qo site. This mechanism, that has been named slowed electron transfer (SET) [159], modulates respiratory electron routing and hypoxic redox signaling [31,160] (Fig. 4D and Table 2).
j) MAO-A and MAO-B are flavin-dependent enzymes anchored to the OMM. They catalyze the oxidative deamination of monoamines, such as serotonin, dopamine, epinephrine, norepinephrine, phenylethylamine and tyramine, and producing the corresponding aldehydes, NH_4_^+^, and H_2_O_2_ [161]. MAO-A and B share ∼70% of their structure. They show some redundancy at the reaction level, but MAO-A preferentially oxidizes serotonin, while MAO-B prefers β-phenylethylamine. Dopamine, epinephrine, norepinephrine and tyramine can be deaminated by both enzymes [162].

Overall, mtROS generation is governed by the interplay among substrate supply, electron transport directionality, ΔΨmt, and respiratory complex organization, with distinct mechanisms: FET, RET or SET, selectively engaging different sites and regulatory pathways to shape cellular redox responses. Thus, in order to provide researchers with the appropriate tools to build up their own workflows, we have designed Table 3, in which each type of mechanism is classified as per the type of inhibitors to which it is sensitive. In this way, each mechanism can be seen as the specific collection of determined features (aka, sensitivity to inhibitors), which will help the readers classify the mtROS mechanism under investigation.

**Table 3:** Features of each mitochondrial ROS production mechanism.

	Response to mitochondrial drugs								mtROS generation pathway
	Rotenone (CI blocker)	Antimycin A (CIII blocker)	FCCP (or other uncoupler)	Myxothiazol (CIII Q_0_ site blocker)	Malonate or dimethylmalonate (CII blocker)	Atpenin A5 (CII blocker)	OGDH, BCODH and PDH inhibitors	NCLX or Na^+^ phospolipid blockers	
**mtROS production**	↑	↓							FET (CI)
	↑	↑		↓					FET (CIII, Q_0_)
	↓		↓		↓	↓			RET
	=		↑/=	↑/=	↓	=			CII-FET ROS
	↓/=		↑/=	↑/=	↓	↓			CII-RET ROS
	↓/=		↑/=	↑/=	↑/=		↓		TCA DH ROS
	↓		↑/=	↓	↓			↓	SET

#### 4.1.1 Factors influencing mtROS production

In the recent years, a number of factors have been observed to influence the production of mtROS. In the next lines, we provide an overview of some relevant factors that in our experience have a major impact on influencing their generation:

1) Ca^2+^ and Na^+^: Ca^2+^ and, in recent years, Na^+^ have emerged as important regulators of mitochondrial (patho)physiology. Current evidence indicates that mitochondrial Na^+^/Ca^2+^ can modulate mitochondrial redox signaling through three principal mechanisms:
a) Excessive mitochondrial Ca^2+^ accumulation induces unspecific mtROS production, whereas alleviating Ca^2+^ overload by inducing its efflux limits ROS generation, which can occur by manipulating NCLX levels [31,163,164]. This triggers mitochondrial permeability transition pore (mPTP) opening, swelling, cyt *c* release, uncoupling, excessive mtROS production, and apoptotic cell death [165,166].
b) Mitochondrial Na ^+^ can directly stimulate mtROS production through SET. This signaling pathway is essential for immunometabolism and cell signaling [[167], [168], [169], [170], [171]], cell death [172], inflammation [173], cardiac metabolism [174,175] and cancer progression [176].
c) Elevated cytosolic Na ^+^ can indirectly increase oxidative stress by diminishing mitochondrial antioxidant capacity. In failing cardiomyocytes, increased cytosolic Na^+^ enhances NCLX activity and lowers steady-state mitochondrial Ca^2+^, which decreases activation of Ca^2+^-dependent TCA-cycle dehydrogenases and NADH production, impairing regeneration of the NADPH pool that sustains antioxidant systems [177,178]. This pathway does not exclude b) and may, in fact, act as its amplifier.
2) Supercomplexes: These structures create partially segmented CoQ and cyt *c* pools that enhance electron transfer efficiency and limit mtROS generation [179,180]. Supercomplexes have been involved in two mtROS production mechanisms, SET and FET:
a) SET induces constraints on CoQ diffusion. This preferentially affects CII to CIII electron transfer, while sparing CI to CIII activity [31].
b) Supercomplexes produce less ROS than free complexes *in vitro* [28,180] and *in vivo* [26]. Tight CIII-CIV association within respirasomes, mediated by the supercomplex assembly factor (SCAF)-1, improves NADH oxidation and O_2_ consumption via FET while suppressing ROS [28], which may be due to localized substrate channeling and/or distinct lipid–protein microenvironments [181].

Collectively, this perspective provides a conceptual framework to understand how mtROS production can be influenced by Na^+^, Ca^2+^ and supercomplexes, helping the reader to further dissect the required workflow for their experimental setting.

### 4.2 mtROS signaling

Mitochondrial signaling refers to regulated outputs from mitochondria that influence cytosolic, nuclear, organellar, and intercellular processes. This emphasizes that mitochondria can produce signals under physiological conditions and that these signals are decoded by specific molecular circuits [182]. The concept has grown from early observations that mitochondria control cellular redox state and Ca^2+^ homeostasis to a modern view in which mitochondria act as information processors. They integrate inputs such as electron flow, O_2_ availability, substrate supply, matrix redox balance, and Ca^2+^ load, and convert these inputs into outputs that tune metabolism, gene expression, immune function, and tissue physiology. A major advance over the last two decades is the recognition that mitochondria can generate H_2_O_2_ signals and that these signals can propagate to the other cellular compartments through defined pathways (e.g., NRF2 programs).

H_2_O_2_ is an essential redox signaling molecule, with little toxicity at physiologically relevant concentrations. H_2_O_2_ production and removal are tightly controlled in all cells and compartments. Its levels range from low, physiological amounts to intermediate, signaling concentrations, and can rise further to mediate toxic processes. It is formed continuously by mitochondrial and non-mitochondrial systems, and the physiological intracellular pools have been claimed to typically be in the low nanomolar range [183]. Despite that H_2_O_2_ can freely diffuse across membranes, its transport can be facilitated by aquaporins, which create gradients between cellular compartments and between the intra- and extracellular space [12]. In parallel, H_2_O_2_ signaling kinetics implies a relay-based system. In this, only the peroxide pulses that exceed local scavenging capacity or that reach specific sensor proteins (with their specific sensitivity) will propagate downstream.

All these modes or regulation of H_2_O_2_ signaling create thresholds, pulse-duration dependence, and the possibility of oscillatory or switch-like behavior. In practice, cells can tune signaling by changing antioxidant capacity, localizing ROS sources, or modulating PRX/TRX recycling. Such tuning is a powerful way to convert bioenergetic/metabolic states into discrete signaling outcomes [[184], [185], [186]]. In this section, we highlight several relevant H_2_O_2_ signaling which have undergone a prominent advance during the last years and in which our laboratories have been implied.

#### 4.2.1 H_2_O_2_ signaling amplification by ROS induced ROS release (RIRR)

RIRR is a classical way by which mitochondrial (and non-mitochondrial) H_2_O_2_ signals are amplified to produce further H_2_O_2_, assuring the reaching of the target molecule. The classical RIRR mechanisms used to encompass three main pathways: 1) mPTP-dependent RIRR, wherein ROS-triggered pore opening causes ΔΨmt collapse and increased CI/III-derived O_2_^•-^ production; 2) inner membrane anion channel (IMAC)-dependent (cyclosporine A (CsA)-insensitive) RIRR, which activates alternative ion channels; and 3) mitochondrial Ca^2+^- H_2_O_2_ crosstalk, where impaired Ca^2+^ extrusion and its concomitant elevation in the matrix amplify H_2_O_2_ production [187,188]. 1) and 3) in combination appear to have a role when transiently “flickering” by mPTP serve in Ca^2+^ signaling [[188], [189], [190]].

A landmark study identified a distinct ROS amplification pathway: H_2_O_2_ triggered phosphorylation of CII flavoprotein (FpSDH) at tyrosine 604 by mitochondrial FGR kinase (FGR-RIRR) [191]. This enhances CII activity and promotes supercomplex remodeling, generating a secondary H_2_O_2_ signal that reinforces FGR activation and phosphorylation of FpSDH in a positive feedback loop. Unlike mPTP-dependent RIRR, FGR-RIRR does not require pore opening, targets a metabolic enzyme rather than pore-forming proteins and maintains, or even hyperpolarizes, initial ΔΨmt while reorienting electron flux [192].

Genetic evidence demonstrates FGR-RIRR is essential for adaptive responses to metabolic stress, as it works as a CI deficiency compensation mechanism [141,191] and as the coordinator of T cell metabolic switching from oxidative to glycolytic metabolism [192]. FGR-RIRR also has a role in innate immune physiology. In macrophages responding to live bacterial pathogen-associated molecular patterns, recognition triggers phagosomal NOXs-derived H_2_O_2_ that activates FGR [193]. Then, FGR-RIRR mediates a Toll-like receptor and NLRP3 inflammasome activation, establishing it as an immunological-metabolic checkpoint that adjusts innate immunity to bacterial viability. Beyond macrophages, FGR-RIRR regulates neutrophil behavior at sites of inflammation. Four-dimensional live-cell imaging studies identified FGR as a critical driver of a pathogenic neutrophil state: a large, sessile, endothelium-embracing phenotype associated with vascular inflammation and tissue injury [194].

In concomitance with facilitated diffusion by aquaporins and filtering by relay mechanisms, RIRR provides a mechanistic basis for threshold behavior: small H_2_O_2_ elevations may trigger controlled relief-valve events. In tissues with high mitochondrial content, such as heart and muscle, this RIRR thresholding may be especially relevant during workload transitions and reperfusion.

RIRR implies that mitochondria are not just point sources but can behave as excitable elements, producing spatiotemporal patterns of H_2_O_2_. Such patterns may coordinate local activation of redox-sensitive enzymes, influence Ca^2+^ microdomains, and modulate cytoskeletal or membrane-proximal signaling. This adds a ‘systems biology’ layer to mitochondrial signaling, local chemistry becomes network dynamics.

#### 4.2.2 Mitonuclear communication

Mitochondria influence nuclear gene expression through retrograde signaling. A key theme is that mitochondria report the state of redox balance and metabolism to the nucleus to coordinate antioxidant defenses, metabolic pathway usage, mitochondrial biogenesis, and quality control. Several pathways contribute: redox-regulated transcription factors (e.g., NRF2, NF-κB or HIF), kinase signaling networks, and stress-response programs that respond to mitochondrial proteostasis or membrane potential perturbations.

Partial inhibition of mitochondrial ATP synthase in intestinal epithelium induces an anti-inflammatory program via H_2_O_2_-dependent NF-κB activation and promotes functional M2 macrophage polarization [195]. Quenching H_2_O_2_ *in vivo*, with a mitochondria-targeted antioxidants (see section 2.3 for more information), decreased NF-κB activation and diminished the protective phenotype, indicating that mtROS are upstream and necessary in this tissue defense axis. Notably, NF-κB and ROS engage in bidirectional regulation. NF-κB target genes can both lower H_2_O_2_ (via antioxidant induction) and increase H_2_O_2_ (via pro-oxidant enzymes). How H_2_O_2_ can either stimulate or inhibit NF-κB depends on topology and location of the redox event [196].

A second paramount example of intercellular H_2_O_2_ mediated signaling was observed in astrocytic/neuronal communication. The overexpression of mitochondrial-tagged catalase in astrocytes decreases H_2_O_2_ production in these cells during adulthood, which led to alterations in the utilization of the pentose-phosphate pathway and GSH metabolism. These led to profound changes in mouse behavior, due to neuron alteration, since these are modulated by the redox status of astrocytes [197]. Reducing mtROS in neurons does not alter motor coordination under basal conditions, but protects against motor discoordination induced by a pro-oxidant neurotoxin [198]. By contrast, abrogation of mtROS in astrocytes does not confer protection against this insult, indicating that the functional consequences of mtROS modulation critically depend on the cellular source from which they arise [47].

Mitochondrial H_2_O_2_ signaling has been proposed as part of O_2_ sensing pathways. These include the activation of the hypoxia inducible factors (HIFs), that are transcription factors regulating metabolic reprogramming, immune responses and cell death pathways, among others [198]. Hypoxia induces a conformational transition of mitochondrial CI which leads to lower H^+^ pumping by this complex [199]. The concomitant matrix acidification mobilizes Ca^2+^ from Ca^2+^-phosphate precipitates. The resulting increase in matrix Ca^2+^ activates the NCLX, promoting Na^+^ entry into the matrix, that interacts with IMM phospholipids [200] to reduce membrane fluidity and restrict the diffusion of CoQ specifically between CII and CIII. This selective alteration in electron carrier mobility enhances O_2_^•-^ generation at CIII, thereby initiating H_2_O_2_ signaling during hypoxia [31]. These redox changes activate downstream cellular responses to hypoxia, establishing Na^+^ influx as a second messenger necessary for acute and chronic (HIF-dependent) hypoxic signaling [160,175,201].

Mitonuclear communication is inherently bidirectional: nuclear programs regulate mitochondrial protein expression, antioxidant enzymes, and dynamics; mitochondria report back through redox and metabolic signals. This creates feedback loops that can stabilize physiology or, when misregulated, sustain chronic stress states. Indeed, substitution of mtDNA alone is sufficient to induce profound and persistent differences in mitochondrial function, including alterations in proteostasis, respiratory chain activity and H_2_O_2_ generation. These H_2_O_2_ changes were associated with divergent systemic phenotypes encompassing insulin signaling, adiposity, telomere dynamics and age-related mitochondrial dysfunction, ultimately producing marked differences in healthspan and longevity between strains [202]. mtDNA-derived H_2_O_2_ differences, reflecting mito-nuclear communication, are not functionally neutral. Instead, they shape cellular and organismal physiology through mito-nuclear genetic compatibility.

### 4.3 mtROS and pathology

Altered levels of mtROS have been associated with a wide range of pathological conditions, either as causal factors or as secondary contributors to disease progression. In this subsection, we discuss several pathological scenarios that we have investigated extensively over recent years and for which increased mtROS production has been causally linked to disease development. In Section 4.3.1, we describe several molecular pathways that are either triggered or amplified by mtROS generation and examine their contribution to pathological processes. Subsequently, in Section 4.3.2, we present two representative examples of metabolic disorders—β-cell dysfunction and metabolic dysfunction-associated steatotic liver disease (MASLD)—in which mtROS production plays a direct pathogenic role.

Through these examples, we aim to provide the reader with a concise overview of the importance of mtROS in disease. Furthermore, these case studies illustrate the translational relevance of accurately measuring redox events and designing rigorous experimental workflows capable of identifying the specific mechanisms underlying pathological ROS production.

#### 4.3.1 mtROS in pathology-associated pathways

The transition from physiological ROS-mediated signaling and homeostasis to a pathological ROS-driven state can occur when mtROS production exceeds the buffering capacity of mitochondrial antioxidant systems. However, recent evidence has demonstrated that the contribution of mtROS to disease extends beyond the classical imbalance between ROS production and antioxidant defenses that results in nonspecific oxidative damage. Indeed, both increases and decreases in mtROS within the physiological range can promote disease by activating signaling pathways at inappropriate times or in inappropriate cellular contexts. Although these pathogenic processes ultimately originate from ROS-mediated modifications of macromolecules, their biological consequences vary considerably depending on the ROS species involved, the affected tissue, the molecular targets modified, and the downstream pathways engaged.

^•^OH can directly react with mtDNA, generating oxidative lesions. Because mtDNA lacks protective histones and possesses relatively limited DNA repair capacity, oxidative damage accumulates rapidly, impairing the expression of ETC subunits and establishing a vicious cycle of mitochondrial dysfunction and further ROS production [203,204]. Likewise, mtROS promote the peroxidation of inner mitochondrial membrane lipids, particularly cardiolipin, and this oxidative damage compromises essential respiratory functions in multiple disease contexts [205,206].

Mitochondrial proteins are also highly susceptible to oxidative damage because ROS are generated in close proximity to the ETC and because many mitochondrial proteins contain oxidation-sensitive amino acid residues, such as cysteine and methionine, as well as redox-sensitive prosthetic groups, including iron–sulfur (Fe–S) clusters [207]. Oxidative modification of metabolic enzymes, including aconitase and ETC complexes, impairs enzymatic activity and directly links ROS accumulation to reduced mitochondrial oxidative capacity [[208], [209], [210]].

Seminal studies revealed that mtROS-induced oxidation of mtDNA enhances its activity as a damage-associated molecular pattern (DAMP), thereby promoting inflammatory signaling [[211], [212], [213]]. Under conditions of elevated mtROS production and mitochondrial damage, oxidized mtDNA can leak into the cytosol. Cytosolic double-stranded mtDNA is recognized by cyclic GMP–AMP synthase (cGAS), which generates cyclic GMP–AMP (cGAMP). cGAMP subsequently activates STING, leading to activation of IRF3 and NF-κB signaling and the induction of type I interferons (IFNs) and pro-inflammatory cytokines, respectively [214]. Consistent with this mechanism, reduced expression of mitochondrial transcription factor A (TFAM) is sufficient to increase cytosolic mtDNA abundance and induce a type I IFN response together with the expression of IFN-stimulated genes in heterozygous *Tfam* models [215]. This increase in mtDNA release is accompanied by the formation of fewer but larger mitochondrial nucleoids and by mitochondrial hyperfusion.

The pro-inflammatory effects of oxidized mtDNA extend beyond activation of the cGAS–STING pathway. Oxidized mtDNA can also activate Toll-like receptor 9 (TLR9), apparently more efficiently than non-oxidized mtDNA in dendritic cells [216] Consistent with this observation, mtDNA has been shown to engage TLR9 within the lysosomes of DNase II-deficient hearts [217]. Furthermore, mtDNA-mediated activation of TLR9 induces severe inflammatory mitochondrial myopathy through disruption of mitochondrial fusion in skeletal muscle [218,219]. Consequently, mtROS influence multiple interconnected processes, including mtDNA oxidation, mitochondrial fusion and fission dynamics, mitophagy, mtDNA release, and TLR9-dependent inflammatory signaling.

Several studies have demonstrated the pathological relevance of circulating or extracellular mtDNA in TLR9-dependent inflammatory disorders, including rheumatoid arthritis, atherosclerosis, acute liver injury, and *Streptococcus pneumoniae* infection [220]. For example, experimental vascular injury in rats increases extracellular mtDNA levels, resulting in lung tissue damage through a TLR9-dependent mechanism [221]. Similarly, elevated circulating mtDNA concentrations in patients with non-alcoholic steatohepatitis have been associated with TLR9 activation and exacerbation of inflammatory responses [222].

A third major mechanism through which mtROS contribute to pathology involves the activation of regulated cell death pathways. mtROS have been causally implicated in multiple forms of regulated cell death, including apoptosis, ferroptosis, and necroptosis, where they often function as upstream execution signals rather than as secondary byproducts. Excessive mtROS can trigger intrinsic apoptosis by promoting cytochrome *c* release and caspase activation. This mechanism has been clearly demonstrated in TNF-α-induced apoptosis, in which ROS accumulation precedes mitochondrial membrane depolarization, cytochrome *c* release, and nuclear condensation [223,224].

mtROS also play a central role in the initiation and amplification of ferroptosis by linking mitochondrial metabolism to iron-dependent lipid peroxidation. Increased mitochondrial respiration and enhanced TCA cycle activity promote electron leakage from CI and CIII, resulting in elevated O_2_^•-^ and H_2_O_2_ production. The original identification of ferroptosis as a ROS- and iron-dependent form of regulated cell death established oxidative stress as a critical execution signal [225]. Subsequent studies demonstrated that mitochondrial metabolism is a major determinant of ferroptotic susceptibility, as pharmacological or genetic suppression of mitochondrial respiration markedly attenuates ferroptosis, whereas enhanced OxPhos activity increases cellular sensitivity [48].

Similarly, mtROS have emerged as important amplifiers of necroptotic signaling, particularly within the TNFα-induced RIPK1/RIPK3 pathway. Experimental evidence indicates that mtROS promote RIPK1 autophosphorylation and subsequent RIPK3 recruitment, establishing a positive feedback loop that reinforces necrosome assembly and activation [226]. Earlier studies in L929 cells demonstrated that TNFα-induced ROS production depends on both RIPK1 activity and mitochondrial respiration and that ROS scavengers can attenuate necrotic cell death, suggesting an upstream role for mtROS in this process [227]. These mtROS are thought to arise, at least in part, from enhanced mitochondrial metabolism downstream of RIPK3 activation and contribute both to oxidative modification of necroptotic signaling components and to the cellular damage characteristic of necroptosis. Although the requirement for mtROS varies among cell types, these observations support a model in which mitochondrial oxidative stress acts as an upstream amplifier of necroptotic signaling.

Another important pathological consequence of mtROS accumulation is the disruption of mitochondrial bioenergetics through oxidative damage to components of energy metabolism and the ETC. ROS-mediated oxidative modifications can inactivate key metabolic enzymes, including Fe–S cluster-containing dehydrogenases such as α-ketoglutarate dehydrogenase, as well as ETC CI, CII, and CIII. These alterations impair electron transfer, reduce coupling efficiency, and compromise ATP production. Early biochemical studies demonstrated that mtROS directly inactivate Fe–S cluster enzymes and ETC components, resulting in diminished respiratory capacity and reduced ATP synthesis [228,229].

#### 4.3.2 mtROS in metabolic pathologies

Alterations in mtROS levels have been mechanistically linked to both β-cell dysfunction and metabolic dysfunction-associated steatotic liver disease (MASLD), two highly prevalent metabolic disorders associated with obesity. Interestingly, independent studies have demonstrated that not only excessive mtROS production, but also excessive antioxidant activity leading to abnormally low mtROS levels, can contribute to the development of these pathologies. In this section, we review the evidence linking alterations in mtROS homeostasis to metabolic disease.

a) β-Cell dysfunction. Pancreatic β-cells possess one of the lowest antioxidant capacities among mammalian cell types, reflecting the important signaling role of mitochondria-derived ROS in β-cell proliferation and insulin secretion. However, sustained hyperglycemia can elevate ROS levels beyond the physiological range, leading to β-cell dedifferentiation and ultimately cell death. Increased mtROS production during chronic hyperglycemia has therefore been proposed as a central contributor to the pathogenesis of diabetes [230]. Because mitochondria are essential for glucose-stimulated insulin secretion (GSIS), mitochondrial damage directly compromises β-cell function and insulin release [231]. Furthermore, chronic hyperglycemia-induced mtROS suppress insulin gene expression by impairing the activity of the transcription factor MafA [232]. Consistent with the importance of oxidative stress in β-cell pathology, activation of the NRF2 pathway protects pancreatic β-cells from oxidative damage in diabetic mouse models [233].

Oxidation of cardiolipin and other mitochondrial phospholipids by mtROS destabilizes the inner mitochondrial membrane, promotes cytochrome *c* release, and activates intrinsic apoptotic pathways in insulinoma cell lines and isolated pancreatic islets [234]. In parallel, hyperglycemia- and lipotoxicity-induced mtROS impair mitochondrial ATP production, reducing the ATP/ADP increase required for closure of ATP-sensitive potassium channels and the subsequent Ca^2+^ influx that triggers insulin exocytosis. As a result, glucose-stimulated insulin secretion becomes progressively compromised, promoting both β-cell dysfunction and loss of β-cell viability. Together, these findings illustrate how pathological mtROS overproduction undermines both the secretory function and survival of β-cells, contributing to the progressive decline in functional β-cell mass observed during diabetes.

Importantly, excessively low mtROS levels can also be detrimental. Gain-of-function of the mitochondrial transporter ABCB10 enhances antioxidant capacity, resulting in reduced H_2_O_2_ levels, decreased mitochondrial respiration, and impaired glucose-stimulated insulin secretion [235] *In vivo* studies further demonstrated that ABCB10 promotes β-cell mass expansion, with proliferating β-cells exhibiting reduced GSIS. These findings suggest that increased ABCB10 activity at inappropriate stages, such as in mature β-cells, may impair mitochondrial function and ROS-dependent signaling pathways required for optimal insulin secretion [235,236].

b) Metabolic dysfunction-associated steatotic liver disease (MASLD). In MASLD, mtROS are considered key drivers of the transition from benign hepatic steatosis to progressive hepatocellular injury. Early studies by Day and James [237] proposed the influential “two-hit” hypothesis, in which insulin resistance and lipid accumulation constitute the first hit, whereas oxidative stress and lipid peroxidation provide the second hit that promotes inflammation, fibrosis, and cell death. Building upon this concept, Sanyal and colleagues [238] demonstrated that mitochondrial dysfunction plays a central role in disease progression, showing that structural mitochondrial abnormalities and impaired β-oxidation lead to fatty acid accumulation, which in turn further enhances ROS production. This oxidative environment is frequently associated with the appearance of megamitochondria and crystalline inclusions, suggesting that ROS-mediated damage contributes directly to ultrastructural alterations in hepatocytes [239]. Moreover, disease severity correlates closely with mitochondrial dysfunction, as mitochondrial respiration is significantly impaired in patients with steatohepatitis, creating a vicious cycle in which defective respiration promotes further ROS production and oxidative damage to mitochondrial DNA [240].

Compelling causal evidence supporting the pathogenic role of mtROS has emerged from genetic mouse models in which mitochondrial antioxidant systems or ETC components are selectively disrupted. For example, deficiency of SOD1 or SOD3 promotes collagen deposition and liver fibrosis under dietary stress [241,242] whereas viral-mediated overexpression of SOD2 attenuates cholestasis-induced liver fibrosis in rats [243]. The importance of lipid-specific oxidative damage is further highlighted by hepatocyte-specific deletion of GPX4, which markedly increases susceptibility to oxidative and metabolic injury [244].

Beyond direct organelle damage, mtROS function as upstream signals that activate innate immune pathways through mtDNA oxidation, mtDNA release, and subsequent activation of both the NLRP3 inflammasome and the cGAS–STING pathway. Under conditions of chronic oxidative stress, mtROS promote the formation of mitochondrial membrane pores, frequently involving the mPTP or BAK/BAX macropores, thereby facilitating the release of oxidized mtDNA into the cytosol. Seminal work by Zhou and colleagues [245] demonstrated that mtROS are both necessary and sufficient for NLRP3 inflammasome activation, largely by promoting the interaction between TRX-interacting protein (TXNIP) and NLRP3, a process that is markedly enhanced under conditions of hepatic lipid overload.

Interestingly, increasing mitochondrial oxidative flux can also be protective in specific metabolic contexts. Deletion of key antioxidant systems in hepatocytes, including the mitochondrial transporter ABCB10 and heme oxygenase 1 (HMOX1), protects against MASLD by enhancing mitochondrial oxidative metabolism and promoting lipid utilization [235,246]. Moreover, nutrient excess induces a state commonly referred to as reductive stress, characterized by elevated NAD(P)H/NAD(P)^+^ ratios in both the cytosol and mitochondria. Because NAD(P)H provides the reducing equivalents required by most antioxidant systems, nutrient overload effectively enhances antioxidant capacity. In support of this concept, Goodman and colleagues [247] demonstrated that expression of a bacterial enzyme capable of oxidizing cytosolic NADH to NAD^+^ protects hepatocytes from obesity-induced insulin resistance. Collectively, these findings support a model in which alleviation of reductive stress restores physiological ROS signaling, increases NAD^+^ availability, and promotes mitochondrial oxidative metabolism, thereby improving insulin sensitivity and metabolic homeostasis.

Overall, these studies highlight that both excessive and insufficient mtROS production can contribute to metabolic disease. Maintaining mtROS within an appropriate physiological range appears essential for preserving normal metabolic signaling, cellular function, and tissue homeostasis.

## 5 The mitochondrial antioxidant systems

The primary objective of this chapter is to provide an overview of the mitochondrial antioxidant pathways that operate within biological systems. A detailed understanding of the mechanisms and interconnected networks responsible for maintaining mitochondrial redox homeostasis is essential for interpreting how cells respond to oxidative stress under both physiological and pathological conditions. These antioxidant systems not only preserve cellular integrity under normal circumstances but also constitute critical adaptive mechanisms that attempt to limit damage during disease.

To achieve this objective, Section 5.1 describes the principal antioxidant pathways operating within mitochondria, whereas Section 5.2 examines the transcriptional programs that regulate and coordinate these antioxidant defenses. Together, these sections provide the reader with a comprehensive understanding of the mechanisms that control mitochondrial redox balance. This knowledge will facilitate the rational design of experimental workflows tailored to specific biological questions, redox processes, and pathological contexts, thereby enabling a more accurate interpretation of mitochondrial redox events.

Together with the knowledge provided in sections 2, 4, Section 5 enables the reader to technically and mechanistically understand the generation of workflows aimed to specifically detect mitochondrial redox events.

### 5.1 Molecular mechanisms of mtROS quenching

**Fig. 5:**
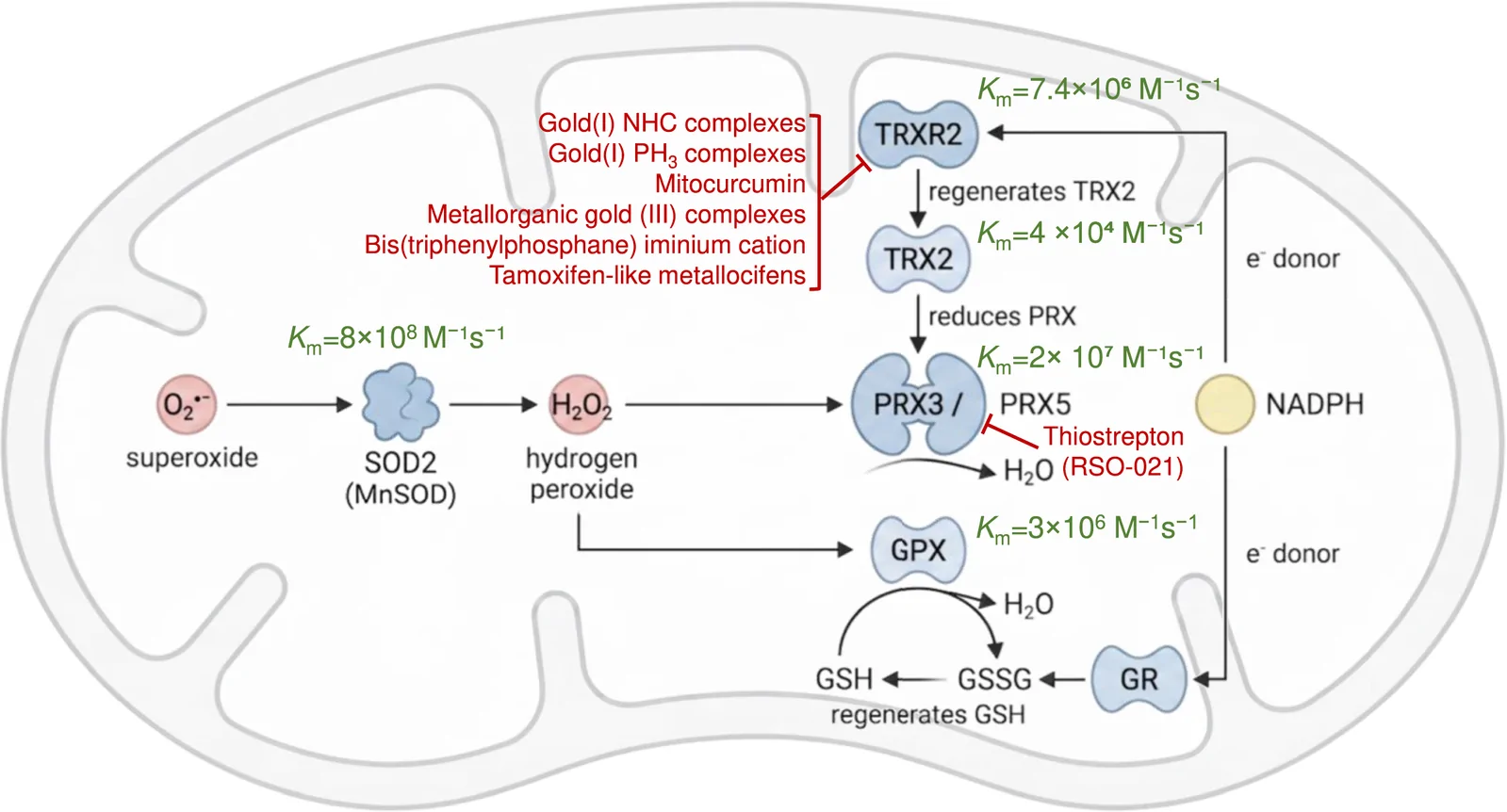
**Major mitochondrial antioxidant systems and their functional interactions.** O_2_•^-^ generated within mitochondria is converted to H_2_O_2_ by manganese superoxide dismutase (SOD2/MnSOD). H_2_O_2_ is subsequently detoxified through two principal antioxidant pathways. In the TRX-dependent pathway, PRX3 and PRX5 reduce H_2_O_2_ to H_2_O using electrons provided by TRX2, which is regenerated by TRXR2 at the expense of NADPH. In the GSH-dependent pathway, GPXs reduce H_2_O_2_ to H_2_O using GSH, generating GSSG. GSSG is subsequently recycled to GSH by GR using NADPH as an electron donor. In parallel, the TRX2/TRXR2 and GRX2 systems regulate the redox state of protein thiols, thereby contributing to redox signaling and protection against oxidative damage. Together, these interconnected antioxidant modules maintain mitochondrial redox homeostasis and control both ROS detoxification and redox-dependent signaling. Only specific inhibitors are shown (in red) [[254], [255], [256]] and the specificity constant (Km) in green [[257], [258], [259], [260], [261]].

A useful operational framework is to organize the mitochondrial antioxidant network (Fig. 5) into functional modules defined by (i) the ROS species they primarily detoxify and (ii) the electron donors they consume. This modular perspective facilitates understanding of how different antioxidant pathways cooperate to maintain mitochondrial redox homeostasis and how dysfunction in individual components can alter both ROS signaling and oxidative stress responses.

a) O_2_^•-^ dismutation in the mitochondrial matrix. SOD2 catalyzes the conversion of O_2_•^-^ into H_2_O_2_, thereby limiting O_2_•^-^-mediated oxidative damage and redox signaling. However, under conditions in which peroxide detoxification is compromised, increased SOD2 activity may elevate local H_2_O_2_ levels [26]. Consistent with its central antioxidant role, SOD2 deficiency accelerates cellular senescence and aging phenotypes [45,248].
b) Peroxide detoxification through the GSH system. GPXs reduce H_2_O_2_ and lipid hydroperoxides using GSH as an electron donor, generating oxidized glutathione (GSSG), which must subsequently be recycled by GR at the expense of NADPH [25,197]. Among the GPX family members, only GPX1 and GPX4 are localized to mitochondria. GPX4 is specialized in the reduction of phospholipid hydroperoxides and plays a crucial role in suppressing ferroptosis, whereas GPX1 exhibits broader substrate specificity and accounts for a substantial proportion of total mitochondrial GPX activity [249]. Deficiency of GPX4 increases susceptibility to ferroptotic cell death, while GPX1 deficiency markedly reduces overall peroxide-detoxifying capacity.
c) Redox buffering of protein thiols: the TRX2/TRXR2 and GRX2 systems. The mitochondrial TRX2 and GRX2 systems reverse protein disulfides and S-glutathionylation, respectively. These reactions protect mitochondrial proteins from oxidative damage while simultaneously functioning as regulatory redox switches that modulate enzyme activity, protein–protein interactions, and apoptotic signaling pathways [[250], [251], [252], [253]].

TRXs constitute a major family of protein-disulfide reductases and serve as electron donors for PRXs, methionine sulfoxide reductases, and other proteins involved in thiol repair and redox signaling [250]. The mitochondrial TRX system is composed primarily of TRX2 and thioredoxin reductase 2 (TRXR2). TRX2 transfers electrons through its conserved C32/C35 active-site cysteines and is maintained in a reduced state by TRXR2 using NADPH [252,253,262]. Although cytosolic TRX1 can translocate to mitochondria under conditions of oxidative stress, TRXR2 displays greater catalytic efficiency toward TRX2 than TRX1 [262,263]. Functionally, TRX2 supports PRX reduction, suppresses ASK1-dependent apoptosis, contributes to respiratory-chain activity, and regulates protein denitrosylation. Accordingly, complete loss of TRX2 results in embryonic lethality [264,265].

The GRX system links NADPH, GR, the GSH/GSSG redox couple, and mitochondrial GRXs. GRX2 catalyzes reversible glutathionylation and deglutathionylation of mitochondrial proteins, including ETC complexes and metabolic enzymes, thereby influencing O_2_^•-^ production, ATP synthesis, and cardiolipin oxidation [266]. These modifications serve both protective and regulatory functions. For example, GRX2 controls the glutathionylation state of CI and uncoupling protein 3 (UCP3), regulates proton leak, and influences mitochondrial fusion, ultrastructure, and bioenergetic function [267,268].

The phenotypes associated with altered GRX2 activity illustrate that antioxidant enzymes do not always function simply as suppressors of oxidative damage. GRX2 overexpression can protect against doxorubicin-induced apoptosis, whereas GRX2 deficiency exacerbates cardiac remodeling, vascular defects, neuroinflammation, and cataract formation [269]. Conversely, partial or complete loss of GRX2 can also trigger adaptive metabolic responses, including protection against diet-induced obesity and fatty liver disease through altered protein glutathionylation, reduced mitochondrial H_2_O_2_ production, and compensatory increases in GPX and TRXR activity [270].

At the mechanistic level, GRX2 regulates mitochondrial bioenergetics through reversible glutathionylation of CI and UCP3, thereby controlling O_2_^•-^ generation, ATP production, and H^+^ leak [266,271]. Loss of GRX2 impairs mitochondrial fusion, ultrastructure, and energetic performance in cardiac tissue, whereas GRX2 overexpression limits cardiolipin oxidation and cytochrome *c* release following doxorubicin exposure [272]. Consequently, GRX2 should be viewed not only as an antioxidant enzyme but also as an important regulator of mitochondrial metabolism.

Adaptive responses associated with GRX2 deficiency further emphasize the context-dependent nature of antioxidant biology. In some high-fat diet models, reduced GRX2 activity promotes beneficial metabolic remodeling characterized by increased respiration, enhanced proton leak, glutathionylation of α-ketoglutarate dehydrogenase, reduced mitochondrial H_2_O_2_ production, and compensatory activation of GPX and TRXR pathways [270,273]. Thus, alterations in GRX2-dependent thiol chemistry may either increase or decrease oxidative burden depending on tissue type, sex, and metabolic context.

In contrast to GRX2, GRX5 serves a distinct function as an Fe–S cluster donor required for the maturation of mitochondrial [2Fe–2S] and [4Fe–4S] proteins. Because mitochondrial Fe–S cluster assembly is also essential for cytosolic, nuclear, and ER-associated Fe–S proteins, defects in GLRX5 compromise diverse cellular processes and have been linked to sideroblastic anemia [274].

d) Peroxide detoxification through the TRX/PRX/sulfiredoxin system. PRX3 and PRX5, maintained by the TRX2/TRXR2 system and supported by sulfiredoxin, constitute the major mitochondrial peroxide-buffering network. This module detoxifies H_2_O_2_, organic hydroperoxides, and ONOO^−^ while simultaneously shaping local H_2_O_2_ signaling and redox-sensitive protein regulation [36,37,275].

PRXs are thiol-dependent peroxidases that reduce H_2_O_2_, alkyl hydroperoxides, and ONOO^−^ [276]. Their catalytic center contains a highly reactive peroxidatic cysteine capable of sensing and transducing H_2_O_2_ signals through the oxidation of downstream effector proteins, including ASK1 and STAT [244]. In 2-Cys PRXs, oxidation by peroxide is followed by disulfide bond formation with a resolving cysteine and subsequent reduction by TRX. Because the peroxidatic cysteine can itself become hyperoxidized to sulfinic or sulfonic forms, PRXs function not only as ROS scavengers but also as regulated peroxide sensors whose temporary inactivation permits localized H_2_O_2_ signaling [37,275,277,278].

Within mitochondria, PRX3 preferentially detoxifies H_2_O_2_, whereas PRX5 exhibits greater activity toward organic hydroperoxides and ONOO^−^ and is additionally found in other cellular compartments [36,37,279]. PRX3 is estimated to remove the majority of mitochondrial H_2_O_2_ and therefore plays a dominant role in matrix peroxide detoxification. Consistent with this function, depletion of PRX3 increases intracellular H_2_O_2_ levels and sensitizes cells to TNF-α-induced apoptosis [280].

During the PRX3 catalytic cycle, H_2_O_2_ oxidizes the peroxidatic cysteine to sulfenic acid, which subsequently forms an intersubunit disulfide reduced by TRX2. Excessive peroxide exposure can drive further oxidation to the sulfinic form, causing reversible loss of peroxidase activity and, in some cases, the formation of higher-order assemblies with chaperone-like properties. Additional oxidation to the sulfonic form is irreversible [37,277,278]. This catalytic cycle enables PRX3 to function simultaneously as a peroxide buffer and a signaling sensor.

Sulfiredoxin restores the activity of hyperoxidized PRX3 by reducing the sulfinic form in an ATP-dependent reaction requiring either GSH or TRX2. Cytosolic sulfiredoxin is imported into mitochondria through an H_2_O_2_-dependent, HSP90-mediated mechanism. The coordinated regulation of sulfiredoxin import and degradation by the Lon protease contributes to circadian oscillations of PRX3 hyperoxidation in multiple mouse tissues [281]. Moreover, p53 promotes necroptosis by inducing PRX3 hyperoxidation and downregulating both PRX3 and sulfiredoxin, thereby limiting the recovery of active PRX3 [282]. Hyperoxidized PRXs, including PRX3, can be detected by immunoblotting using antibodies directed against the conserved hyperoxidized active-site peptide, which recognize both sulfinic and sulfonic forms. The higher molecular weight of PRX3 facilitates its discrimination from PRX1 and PRX2 on immunoblots [282].

Although sulfiredoxin was initially thought to act exclusively on hyperoxidized 2-Cys PRXs, recent proteomic studies suggest that cysteine sulfinylation is widespread and that sulfiredoxin may act on additional protein targets [283]. These observations raise the possibility that reversible protein sulfinylation constitutes a broader regulatory mechanism than previously appreciated.

TRX2 is generally regarded as the principal reductant of mitochondrial PRX3 because GRX2 exhibits substantially lower catalytic efficiency, and PRX5 can be reduced by TRX2 but not by GRX2 [257,284]. Consistent with this model, TRX2 haploinsufficiency or cardiac-specific deletion increases mtROS levels and impairs ATP production, respiratory-chain activity, and cardiac function [49,50].

The activity of PRX3 is further regulated by acetylation at lysine 253289. Mitochondrial SIRT3 deacetylates PRX3, thereby protecting against mitochondrial oxidative damage, apoptosis, and ischemia–reperfusion-induced intestinal injury [285].

PRX5 also plays important protective roles in disease-relevant contexts. Reduced expression or loss of PRX5 increases susceptibility to MPP^+^-induced neurotoxicity and may contribute to the vulnerability of dopaminergic neurons. Furthermore, PRX5 deficiency exacerbates high-fat-diet-induced obesity, hepatic steatosis, inflammation, and hypertriglyceridemia [286]. PRX5 activity is additionally regulated by S-palmitoylation, ABHD10-dependent deacylation, and non-covalent inhibition by itaconate, thereby linking mitochondrial peroxide detoxification to lipid metabolism and innate immune signaling [287,288].

e) Mitochondrial NADPH supply. Because both GSH-dependent and TRX-dependent antioxidant systems consume NADPH, either directly or indirectly, the availability of mitochondrial NADPH frequently constitutes a limiting determinant of antioxidant capacity even when antioxidant enzyme expression remains unchanged [25,47]. Major contributors to mitochondrial NADPH generation include nicotinamide nucleotide transhydrogenase (NNT), isocitrate dehydrogenase 2 (IDH2), mitochondrial malic enzyme, and cytosolic–mitochondrial redox shuttles.

Importantly, these antioxidant modules generate distinct redox signatures. For example, primary impairment of SOD2 typically increases O_2_•^-^-dependent signals, such as MitoSOX fluorescence, with variable effects on bulk H_2_O_2_ levels. In contrast, dysfunction of the GPX, PRX, or TRX systems often results in relatively preserved O_2_•^-^ levels but enhanced H_2_O_2_-dependent oxidation of protein thiols and increased sensitivity to exogenous peroxide [26].

### 5.2 Transcriptional regulation of the mitochondrial antioxidant system

Transcriptional responses reshape mitochondrial antioxidant capacity over timescales ranging from hours to days by altering the expression of antioxidant enzymes, transporters, and metabolic pathways that supply reducing equivalents. These responses involve not only antioxidant enzymes themselves, but also factors regulating GSH synthesis, enzymes responsible for NADPH production, and broader programs that modulate substrate utilization and mitochondrial turnover. As a general principle, transcriptional regulation is likely to contribute to phenotypes that require more than 4–6 h to develop or that are prevented by inhibition of transcription or translation. In contrast, responses occurring within minutes to approximately 2 h generally reflect acute redox switches or metabolic adaptations [25,47].

**Fig. 6:**
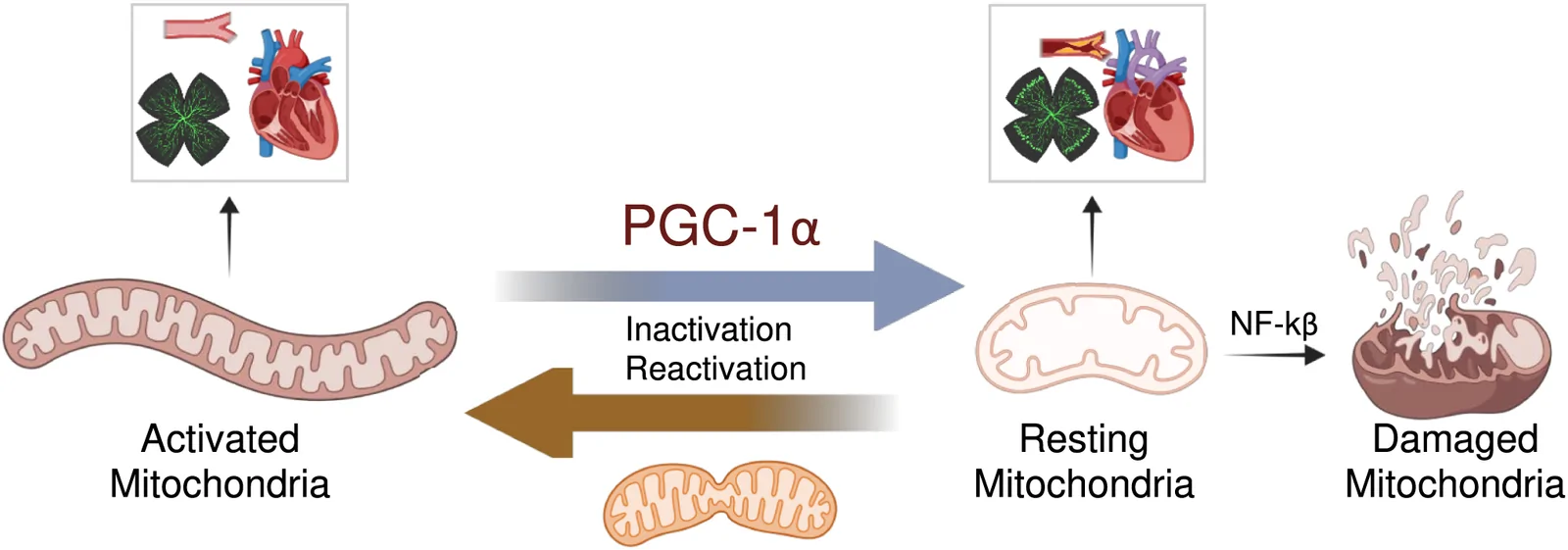
Conceptual scheme of mitochondrial functional states, ranging from a resting condition to activated, superactivated, and damaged states, together with the processes of deactivation and reactivation; the potential role of PGC-1α in the recovery and remodeling of the mitochondrial state is also highlighted.

At this level, antioxidant regulation cannot be separated from metabolic homeostasis. OxPhos is a major cellular source of ROS, and oxidative stress emerges when the balance between ETC-derived oxidants and antioxidant defenses is disrupted. Consequently, transcriptional regulation of mitochondrial antioxidant systems must be interpreted within the broader context of ETC activity, substrate utilization, mitophagy, and the overall metabolic state of the cell (Fig. 6) [289].

The FOXO family of transcription factors provided some of the earliest evidence linking metabolism to antioxidant defense. FOXO1 and FOXO3 are inhibited by insulin and IGF-1 signaling under nutrient-rich conditions, whereas nutrient deprivation activates these factors and promotes the expression of antioxidant genes such as SOD2, thereby coupling metabolic status to mitochondrial redox protection [290].

PGC-1α coactivators further extend this connection by coordinating mitochondrial biogenesis, OxPhos, and antioxidant defense. PGC-1α regulates the expression of genes involved in both limiting mtROS production and detoxifying oxidants, while ROS themselves can positively regulate PGC-1α activity, creating a feedback system that couples oxidative metabolism with antioxidant capacity [291].

PGC-1α-dependent transcriptional programs support multiple aspects of mitochondrial redox homeostasis, including SOD2-mediated dismutation of matrix O_2_^•-^ (mainly through FOXO3a transcriptional program) [292,293], peroxide detoxification through GPX and PRX systems, GSH- and TRX-dependent repair of oxidized proteins, NADPH generation, respiratory-chain organization, mitochondrial uncoupling responses, and mitophagy [294]. This broad regulatory scope is important because mitochondrial redox homeostasis depends not only on peroxide removal but also on limiting electron leakage, maintaining the proton-motive force within a physiological range, repairing oxidized protein thiols, and eliminating irreversibly damaged mitochondria. Thus, PGC-1α influences both the production and detoxification arms of mtROS homeostasis.

At the molecular level, mitochondrial H_2_O_2_ primarily oxidizes reactive cysteine thiolates, generating sulfenic acid intermediates that can either be reduced back to thiols, converted into disulfides, or further oxidized to sulfinic and sulfonic acid species. The GRX/GSH/GR and TRX/TRXR systems reverse many of these modifications, restoring proteins to their reduced state. Within mitochondria, the TXN2/TXNRD2 system plays a particularly important role in both thiol repair and redox signaling [295].

Several mitochondrial mechanisms also limit ROS production before antioxidant detoxification becomes necessary. These include ETC components that improve electron-transfer efficiency under conditions of high metabolic flux, regulatory proteins that restrain respiration during hypoxia, uncoupling proteins (UCPs) that dissipate excessive proton gradients, and OPA1-dependent organization of respiratory complexes within mitochondrial cristae [296]. Such mechanisms demonstrate that transcriptional antioxidant programs are closely integrated with mitochondrial bioenergetic architecture.

When oxidative damage persists, mitochondria become increasingly vulnerable because mtDNA, Fe–S cluster-containing enzymes, respiratory complexes, and membrane lipids accumulate oxidative lesions. Progressive mitochondrial damage can subsequently activate mitophagy, including ROS-sensitive quality-control pathways that selectively eliminate dysfunctional mitochondria and thereby prevent further amplification of oxidant production [297].

The physiological relevance of this coordinated regulation is particularly evident in vascular biology and inflammation. In endothelial cells, PGC-1α-dependent control of mtROS influences angiogenesis and vascular permeability (Fig. 6). PGC-1α levels decrease during hypoxia-associated vascular remodeling and are restored during reperfusion, promoting vascular stability and protection against ischemia–reperfusion injury [298]. In inflammatory settings, NF-κB signaling can suppress PGC-1α expression, thereby reducing oxidative metabolism and limiting antioxidant induction. Failure to restore PGC-1α activity, as observed in certain obesity-associated conditions, may therefore exacerbate tissue damage (Fig. 6) [299].

The role of PGC-1α in cancer is more complex and highly context dependent. In some tumors, increased PGC-1α activity enhances antioxidant capacity and promotes resistance to chemotherapy. In others, it reduces mitochondrial and nuclear DNA damage and suppresses characteristics associated with transformation, senescence, and metastatic progression [300].

A second major transcriptional hub is NRF2. Activation of NRF2 is classically regulated by the redox-sensitive KEAP1 system and induces a broad antioxidant program encompassing genes involved in GSH synthesis, NADPH metabolism, and peroxide detoxification. Early genetic studies demonstrated that Nrf2 deficiency promotes MASLD, whereas hepatic activation of NRF2 through loss of KEAP1 protects against disease progression. These effects are associated with alterations in mitochondrial OxPhos, membrane potential, and ATP production [301].

Subsequent work revealed extensive crosstalk between NRF2 and PGC-1α. These pathways are co-regulated at multiple levels, and NRF2 can induce expression of UCP3, thereby reducing mtROS production. Furthermore, NRF2 activation is associated with enhanced mitochondrial quality control, including regulation of mitophagy, mitochondrial dynamics, and organelle mobility [302]. Consequently, NRF2 should not be viewed merely as a generic antioxidant switch but rather as a broader regulator of metabolic remodeling that influences mtROS production itself.

The interpretation of transcriptional antioxidant responses must therefore take metabolic context into account. For example, in primary vascular endothelial cells, replacing glucose with galactose forces greater reliance on OxPhos and substantially alters FOXO3 and NRF2 signaling. Under these conditions, FOXO3 abundance increases, NRF2 becomes relatively enriched within the nucleus, and the transcriptional response to the redox-active compound diphenyl diselenide [(PhSe)_2_] becomes both stronger and more sustained [303]. Whereas FOXO3 activity is suppressed by AKT signaling in glucose-containing media, it increases approximately 24 h after exposure to galactose, and the antioxidant response induced by (PhSe)_2_ is significantly enhanced under OxPhos-dependent conditions [304].

These observations demonstrate that the same redox-active molecule can elicit distinct transcriptional responses depending on the metabolic state of the cell. More broadly, they underscore that redox-sensitive transcription factors do not operate in isolation. Their activity depends on mitochondrial function, nutrient availability, and the intracellular oxidant burden. Consequently, antioxidant transcriptional responses should be evaluated alongside metabolic parameters rather than being interpreted solely as isolated changes in gene expression.

## 6 Recommended experimental workflows

**Fig. 7:**
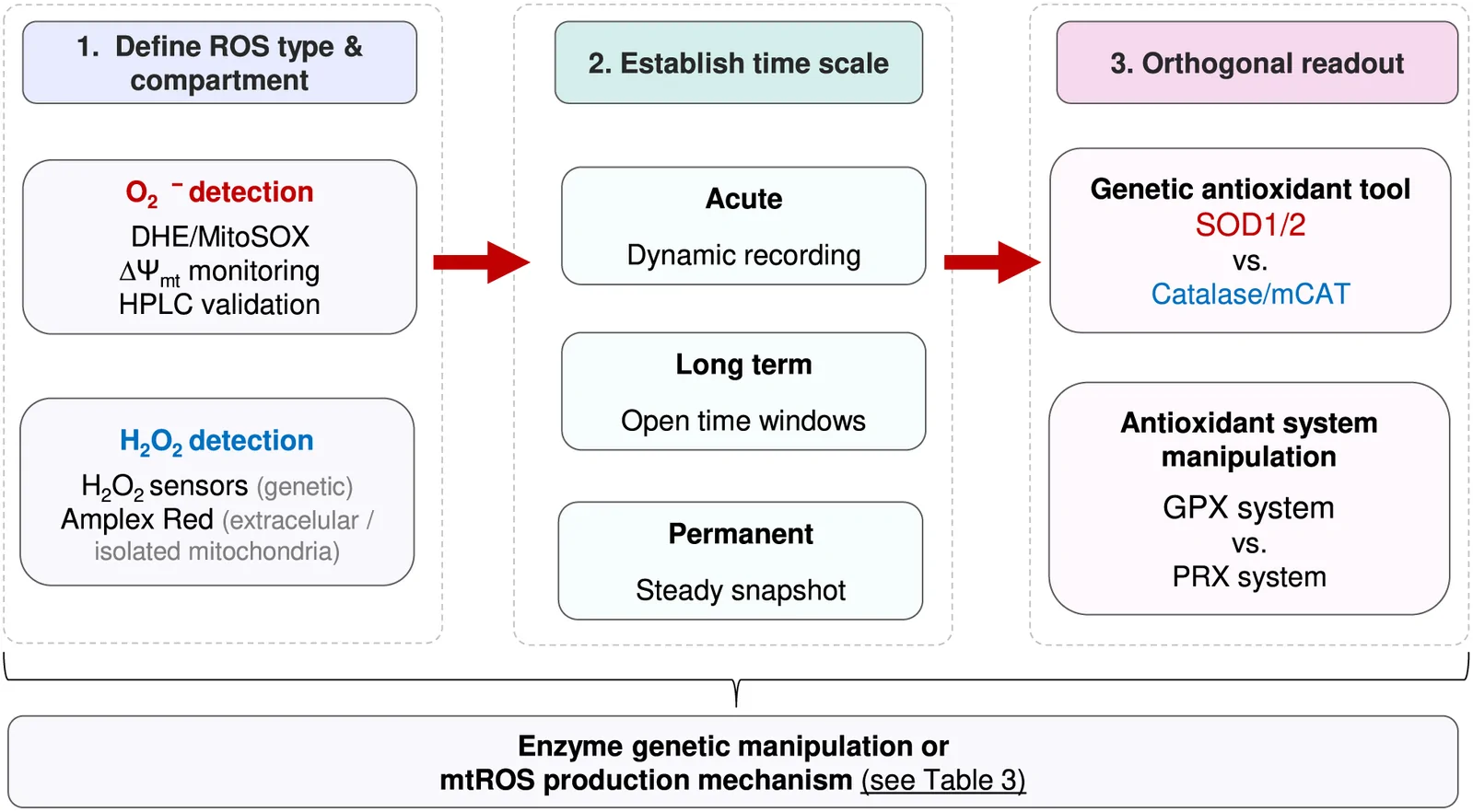
Recommended experimental workflow to measure specific mtROS in a variety of experimental conditions. The type of probes, the controls and the readouts are depicted in different colors and boxes.

As outlined in the Introduction, we define a workflow as the combination of tools, methodologies, and conceptual knowledge required to identify and characterize a specific redox event. In Section 2, we provided an overview of the principal tools and methods available for ROS detection and manipulation. Sections 3, 4 described the major cellular sources of ROS and several examples of their roles in physiology and disease, while Section 5 reviewed the mitochondrial antioxidant systems that shape redox homeostasis. Having established the fundamental concepts within our principal areas of expertise in redox biology, we now present a general recommended workflow (Fig. 7). This, presented as a chart, summarizes how to detect any type of mitochondrial redox event using the best currently available tools, as well as the appropriate methodologies and controls. This workflow is linked to Table 3, which summarizes the necessary mechanistic knowledge to discern mtROS production pathways in a snapshot. In the following lines, we propose a series of hypothetical scenarios, similar to some that we have faced, that researchers may encounter and that illustrate how specific redox events can be rigorously dissected using the workflow in Fig. 7.

### 6.1 Representative workflow 1

Scenario: A treatment induces a redox-dependent adaptive cellular response that is suspected to be mediated by ROS production, and the aim is to dissect the specific redox event responsible for this response.

Workflow: To identify the specific ROS species involved, determine its source, and characterize its kinetics, researchers should follow the strategy outlined in Fig. 7. The first step is to define both the ROS species and the subcellular compartment involved. Because adaptive responses are most commonly mediated by H_2_O_2_ signaling (see Section 4.2), the approaches highlighted in blue in Fig. 7 are recommended. In intact cells, cytosolic genetically encoded H_2_O_2_ reporters such as roGFP2–Tpx1.C169S or HyPer7 are particularly suitable because H_2_O_2_ can diffuse across biological membranes and therefore be detected independently of its site of production (see Section 2.1).

The second step is to establish the temporal profile of ROS production. Since signaling-related H_2_O_2_ generation is often transient, fluorescence should be monitored dynamically following treatment application.

The third step involves the use of targeted antioxidant interventions as orthogonal approaches to identify the ROS source. Genetically encoded catalase is recommended for selective H_2_O_2_ removal (see Section 2.4.1), and compartment-specific targeting of catalase can reveal the location of ROS generation. Similarly, the fluorescent reporter itself can be targeted to different subcellular compartments, thereby providing additional information regarding the site of ROS production.

If mitochondria are identified as the source of ROS, Table 3 should be used to determine the underlying mechanism based on inhibitor-response profiles. For example, if rotenone and antimycin A enhance the ROS signal induced by the treatment, whereas myxothiazol suppresses it, the data are consistent with a FET mechanism. Conversely, if treatment-induced ROS production is abolished by rotenone and, in a separate experiment, by FCCP, the results support a RET mechanism (see Section 4.1). Importantly, no single inhibitor is sufficient to define a specific mtROS mechanism, as most inhibitors affect multiple ROS-generating pathways. For instance, rotenone can inhibit several distinct mechanisms of mtROS production. If ROS originate outside mitochondria, selective inhibitors or genetic perturbations targeting the relevant ROS-producing or antioxidant enzymes should be employed (see Sections 3, 5).

### 6.2 Representative workflow 2

Scenario: Researchers aim to specifically measure mitochondrial O_2_^•-^ production in whole cells derived from a knockout model.

Workflow: In this case, both the ROS species and the subcellular compartment have already been defined. Furthermore, because the experimental comparison involves a constitutive genetic modification, dynamic measurements are not strictly necessary and steady-state assessment is generally sufficient. Consequently, this workflow follows the approaches highlighted in red in Fig. 7, corresponding to O_2_^•-^ detection.

The most widely used tool for measuring mitochondrial O_2_^•-^ is MitoSOX (see Section 2.2). We recommend combining fluorescence microscopy with HPLC-based analysis (Table 1). Microscopy provides spatial information and confirms mitochondrial localization, whereas HPLC enables discrimination of the O_2_^•-^-specific oxidation product, thereby improving specificity. Because mitochondrial accumulation of MitoSOX depends on ΔΨmt, it should always be measured in parallel (Table 1) [29]. As an orthogonal validation strategy, manipulation of SOD2 expression can be used to confirm both the identity and localization of the detected ROS species (see Section 2.4.1 and Fig. 7).

### 6.3 Representative workflow 3

Scenario: A mouse model of disease exhibits increased mtROS levels and the aim to dissect the specific mechanism involved in this redox event.

Workflow: Because mitochondrial inhibitors are generally too toxic for mechanistic studies *in vivo*, detailed investigation of the underlying mtROS pathway should be performed using isolated mitochondria obtained from the tissue of interest. According to Fig. 7, once the mitochondrial origin of ROS has been established, the next step is to select the ROS species to be measured. Two principal approaches are available: (i) measurement of H_2_O_2_ using Amplex Red or (ii) measurement of O_2_^•-^ using DHE (see Section 2.2). In both cases, ROS are measured acutely under controlled experimental conditions.

Given the limitations in probe specificity, the identity of the ROS species should be validated using targeted antioxidant controls. Specifically, Amplex Red measurements should be accompanied by catalase treatment, whereas DHE measurements should include SOD as a specificity control (see Fig. 7) [24,25,240,294,295].

Once these conditions have been established, the precise mechanism of mtROS production can be investigated using the criteria described in Table 3 and Section 4.1. Importantly, any proposed mechanism should satisfy all the experimental requirements listed in the table rather than relying on a single pharmacological intervention.

Whenever possible, researchers should demonstrate that restoration of the relevant redox event to control conditions rescues the biological phenotype, whereas interventions targeting an incorrect ROS species or subcellular compartment do not. In addition, it is important to verify that the experimental intervention does not simply suppress respiration or broadly impair cell viability, as such effects may confound mechanistic interpretation.

### 6.4 Representative workflow 4

Scenario: A cellular model shows higher roGFP fluorescence than its isogenic control and the aim is to dissect the specific redox event underlying this difference.

Workflow: According to Fig. 7, the compartment is already defined and the type of ROS is H_2_O_2_. In parallel, given that a steady snapshot measurement has already been performed, it is neither necessary to establish the time scale. Thus, an orthogonal readout is required and, for that, NAC is applied to the cellular model with the objective of reducing the roGFP signal up to control levels. However, NAC treatment does not have any effect and roGFP signal remains as in untreated cells; whereas cytosol-targeted catalase quenches the whole signal up to control levels.

In a series of subsequent experiments, following the design in Table 3, all mitochondrial drugs produce a parallel increase of decrease in H_2_O_2_ levels, with none of them equalizing the cellular model and its isogenic control. The same results arise after inhibiting other non-mtROS sources.

In this type of scenario, in which two or more antioxidants have differential effects on the ROS signal, the molecular divergence between the cellular model and its control can be found in their antioxidant buffer system. In this case, roGFP is directly and primarily influenced by NAC through GRX/GSH/GR system (Fig. 1), making GRX/GR as the most probable candidates to cause the differences in roGFP signal, rather than a mtROS production mechanism.

## 7 Future perspectives

Future research will move toward a fully integrated and compartment-resolved understanding of mitochondrial redox biology. A major objective will be to identify, under physiologically relevant O_2_ tensions and metabolic conditions, the predominant sources of ROS *in vivo*, the mechanisms that spatially restrict redox signaling, and the contribution of inter-organelle contact sites to ROS propagation and antioxidant buffering. A detailed understanding of the mechanisms responsible for ROS generation will facilitate the development of compounds capable of selectively targeting specific ROS-producing pathways, potentially leading to important clinical advances. In parallel, the implementation of orthogonal experimental strategies that combine genetically encoded biosensors, targeted perturbations, functional rescue approaches, and rigorous bioenergetic controls will be essential for distinguishing causal ROS production from secondary oxidant generation and for accurately characterizing localized redox events. Collectively, these advances will enable the identification of context-specific therapeutic targets capable of modulating physiological redox signaling while minimizing pathological oxidative stress.

## CRediT authorship contribution statement

**Pablo Hernansanz-Agustín:** Writing – review & editing, Writing – original draft, Visualization, Validation, Supervision, Conceptualization. **Juan Pedro Bolaños:** Writing – review & editing, Writing – original draft, Validation, Supervision. **Consuelo Borrás:** Validation, Supervision, Writing – review & editing, Writing – original draft. **Elisa Cabiscol:** Writing – review & editing, Writing – original draft, Validation, Supervision. **Victoria Campuzano:** Writing – review & editing, Writing – original draft, Validation, Supervision. **Laura de Cubas:** Writing – review & editing, Writing – original draft, Validation, Supervision. **José Antonio Enríquez:** Writing – review & editing, Writing – original draft, Validation, Supervision. **Isabel Fabregat:** Writing – review & editing, Writing – original draft, Validation, Supervision. **Gemma Gonfaus-Ortiz:** Writing – review & editing, Writing – original draft, Validation, Supervision. **Elena Hidalgo:** Writing – review & editing, Writing – original draft, Validation, Supervision. **Marc Liesa:** Writing – review & editing, Writing – original draft, Validation, Supervision. **Cristina Mas-Bargues:** Writing – review & editing, Writing – original draft, Validation, Supervision. **Antonio Martínez-Ruiz:** Writing – review & editing, Writing – original draft, Validation, Supervision. **María Monsalve:** Writing – review & editing, Writing – original draft, Validation, Supervision. **Rubén Quintana-Cabrera:** Writing – review & editing, Writing – original draft, Validation, Supervision. **Sergio Rius-Pérez:** Writing – review & editing, Writing – original draft, Validation, Supervision. **Juan Sastre:** Writing – review & editing, Writing – original draft, Validation, Supervision. **Antonio Zorzano:** Writing – review & editing, Writing – original draft, Validation, Supervision.

## Declaration of competing interest

The authors declare no competing interests.

## Data Availability

No data was used for the research described in the article.
